# Initiation of genomics-assisted breeding in Virginia-type peanuts through the generation of a *de novo* reference genome and informative markers

**DOI:** 10.3389/fpls.2022.1073542

**Published:** 2023-01-27

**Authors:** Cassondra S. Newman, Ryan J. Andres, Ramey C. Youngblood, Jacqueline D. Campbell, Sheron A. Simpson, Steven B. Cannon, Brian E. Scheffler, Andrew T. Oakley, Amanda M. Hulse-Kemp, Jeffrey C. Dunne

**Affiliations:** ^1^ Department of Crop and Soil Sciences, North Carolina State University, Raleigh, NC, United States; ^2^ Institute for Genomics, Biocomputing, and Biotechnology, Mississippi State University, Mississippi State, MS, United States; ^3^ United States Department of Agriculture–Agricultural Research Service (USDA–ARS), Corn Insects and Crop Genetics Research Unit, Ames, IA, United States; ^4^ United States Department of Agriculture–Agricultural Research Service Genomics and Bioinformatics Research Unit, Stoneville, MS, United States; ^5^ United States Department of Agriculture–Agricultural Research Service Genomics and Bioinformatics Research Unit, Raleigh, NC, United States

**Keywords:** genome, genotyping, introgression, legume, peanut, markers, plant breeding, cultivar development

## Abstract

**Introduction:**

Virginia-type peanut, *Arachis hypogaea subsp. hypogaea*, is the second largest market class of peanut cultivated in the United States. It is mainly used for large-seeded, in-shell products. Historically, Virginia-type peanut cultivars were developed through long-term recurrent phenotypic selection and wild species introgression projects. Contemporary genomic technologies represent a unique opportunity to revolutionize the traditional breeding pipeline. While there are genomic tools available for wild and cultivated peanuts, none are tailored specifically to applied Virginia-type cultivar development programs.

**Methods and respective results:**

Here, the first Virginia-type peanut reference genome, “Bailey II”, was assembled. It has improved contiguity and reduced instances of manual curation in chromosome arms. Whole-genome sequencing and marker discovery was conducted on 66 peanut lines which resulted in 1.15 million markers. The high marker resolution achieved allowed 34 unique wild species introgression blocks to be cataloged in the *A. hypogaea* genome, some of which are known to confer resistance to one or more pathogens. To enable marker-assisted selection of the blocks, 111 PCR Allele Competitive Extension assays were designed. Forty thousand high quality markers were selected from the full set that are suitable for mid-density genotyping for genomic selection. Genomic data from representative advanced Virginia-type peanut lines suggests this is an appropriate base population for genomic selection.

**Discussion:**

The findings and tools produced in this research will allow for rapid genetic gain in the Virginia-type peanut population. Genomics-assisted breeding will allow swift response to changing biotic and abiotic threats, and ultimately the development of superior cultivars for public use and consumption.

## 1 Introduction

Peanut (*Arachis hypogaea* L., 2n=4x=40) is a staple crop that is cultivated and consumed globally as a high-quality source of protein and oil. Peanut is a nutritional ‘superfood’ because it contains healthy monounsaturated oil, protein, macronutrients, micronutrients, vitamins and bioactive peptides, all of which are important for human health (Davis & Dean, 2016). When compared to tree nuts and meats, peanuts offer an inexpensive source of protein, making it more accessible for human consumption (Economic Research Service U.S. DEPARTMENT OF AGRICULTURE, n.d). Peanut is a sustainable source of protein as it is able to grow in poor, sandy soils. It is also able to fix nitrogen, and it requires less water than most tree nuts (Mekonnen & Hoekstra, 2011). In the United States (US), nearly 1.6 million acres of peanuts are planted annually across three production regions: the Southeast, Southwest, and Virginia-Carolinas regions (Acreage, 2021). Of the four market types of US peanuts, Runner-type peanuts occupy the largest acreage and are grown in the Southeast. Virginia-type peanuts occupy the second largest acreage and are primarily grown in the Virginia-Carolinas region. Runner-type peanuts and Virginia-type peanuts both belong to the subspecies *hypogaea* and the botanical variety *hypogaea*. Virginia-type peanuts are known for their brightly-colored hulls and large kernel size, which is why they are preferred for gourmet snacks and in-shell products.

**Figure 1:**
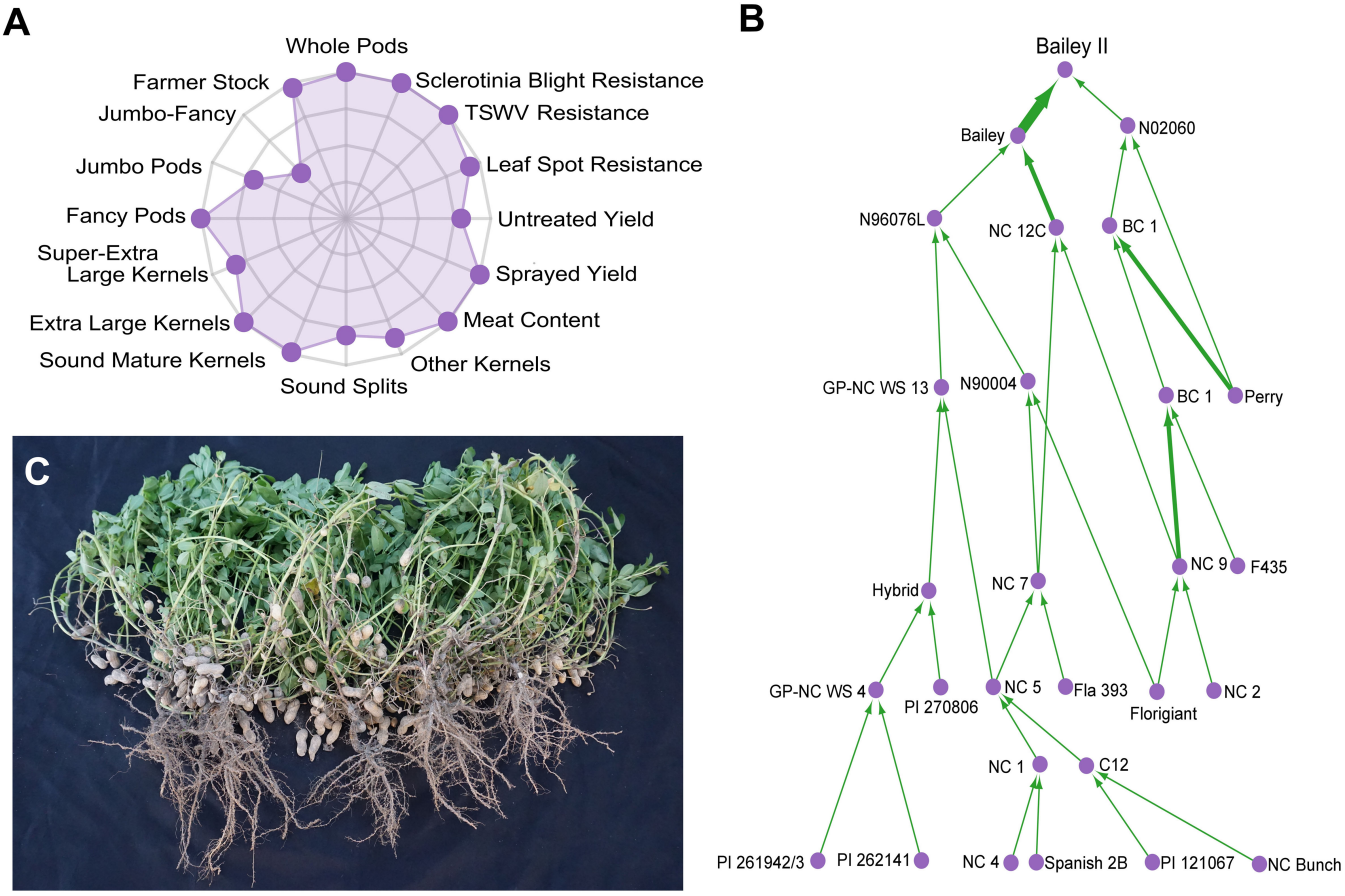
Characterization of the Virginia-type peanut cultivar ‘Bailey II’. **(A)** Radar chart showing the agronomic properties of Bailey II proportionally ranked against 200 modern Virginia-type cultivars and breeding lines from North Carolina State University. Data can be further explored at ‘http://go.ncsu.edu/peanut-breeding’. Possible ranks are zero (center) to ten (outer axis). **(B)** Bailey II pedigree. Individuals are represented as purple circles. The green arrows are weighted by parental contribution; a back-cross will have a wider arrow than a standard cross. PI 262141 is the *A. cardenasii* line [GKP 10017] that was used in the historic introgression projects at North Carolina State University and Texas A&M University in the 1960s. **(C)** Photograph of five Bailey II plants grown with standard seed spacing and cultural practices for North Carolina.

Since its formation in 1929 (Isleib, 2016), North Carolina State University’s (NCSU) public Virginia-type peanut breeding program has been the primary contributor of high yielding, disease-resistant Virginia-type peanut cultivars. Cultivar ‘Bailey II’ (PVP - Bailey II, n.d) is the program’s latest release (2017). It is a near-isogenic line to the widely-adopted cultivar ‘Bailey’ (Isleib et al., 2011). Bailey II possesses the high-oleic trait for improved shelf-life (Norden et al., 1987; O’Keefe et al., 1993; Mozingo et al., 2004) and is well suited to replace Bailey in the Virginia-Carolinas region (**Figure 1**). Historically, long-term phenotypic recurrent selection and the utilization of wild species introgressions have been the basis for population improvement. Wild diploid species have high levels of genetic polymorphism and phenotypic variation when compared to cultivated allotetraploids (Stalker, 2017).

NCSU was not the only program working to introgress wild genetics. In the 1960s, both NCSU and Texas A&M University (TAMU) were working to circumvent the ploidy barrier between allotetraploid cultivated peanut and valuable wild diploid species. Two successful introgression routes were utilized: the ‘hexaploid route’ at NCSU (Gregory & Smartt, 1967) and the ‘tetraploid route’ at TAMU (Simpson, 1991). In both programs, *A. cardenasii* Krap. et Greg. GKP 10017 (PI 262141) (2n=2x=20) was used as the wild species introgressed into cultivated peanut. It was well documented that *A. cardenasii* has moderate resistance (Stalker & Beute, 1993) to early leaf spot (*Passalora arachidicola*), and high levels of resistance (Abdou et al., 1974) and (Stalker et al., 1979) to late leaf spot (*Nothopassalora personata*) and root knot nematode (*Meloidogyne arenaria*) (Nelson et al., 1989), all of which cause great economic damage to peanut. Seventeen ‘GP-NC WS’ germplasm lines were selected and released from the NCSU hexaploid route for their pathogen-resistant phenotypes (Stalker and Beute, 1993; Stalker et al., 2002; Stalker and Lynch, 2002). Out of the TAMU tetraploid route, two ‘TxAG’ germplasm lines were released for their resistance to root knot nematode and leaf spot foliar diseases (Simpson et al., 1993). Several *A. cardenasii* introgression blocks exist in cultivated peanut populations as a result of these introgression projects (Bertioli et al., 2021).

Recent research and technological advances offer solutions for increasing the rate of genetic gain and selection efficiency in breeding programs. Marker-assisted selection (MAS) is a promising technology for targeting traits with simple inheritance while genomic selection (GS) can be used to efficiently select for complex traits. There is tremendous opportunity for utilizing MAS to exploit the historic wild species introgression work conducted at NCSU and TAMU. In particular, a high density set of validated markers interspersed within the historic *A. cardenasii* introgressions will enable identification, evaluation, and subsequent selection of introgression blocks with MAS.

Success of GS depends on a consistent, high-throughput, yet economical genotyping pipeline tailored to a specific breeding program (Andres et al., 2020). When considering peanut’s genetic map length of 3,264 centiMorgans and a target of one marker per centiMorgan (Zhuang et al., 2019); this requires approximately 3,250 markers in peanut. The prominent genotyping platform in peanut is the Axiom Arachis2 48K SNP array (Clevenger et al., 2018). However, genotyping of 200 diverse Virginia-type peanut breeding lines, cultivars, and germplasm lines from the NCSU program on this array yielded an insufficient number (1,317) of informative markers to initiate GS after filtering for minor allele frequency and linkage disequilibrium (LD) (Hancock, 2018). To design an effective GS genotyping system for the Virginia-type peanut breeding program, marker discovery using individuals from the target breeding population is needed in conjunction with an appropriate reference genome. While multiple allotetraploid peanut reference genomes are available (Bertioli et al., 2019; Chen X. et al., 2019; Zhuang et al., 2019), none of them are Virginia-type peanuts or carry wild-species introgressions. Developing a Virginia-type peanut-specific reference genome using contemporary long read sequencing on a prominent Virginia-type peanut cultivar will provide the foundation for subsequent marker discovery. Marker information will allow for the design of a mid-density genotyping protocol for GS.

This study developed a contiguous, high-quality, *de novo* peanut reference genome assembly for the most recent Virginia-type peanut cultivar, ‘Bailey II’. The assembly enabled variant discovery and evaluation of genomic relationships for 66 peanut lines that are important to NCSU Virginia-type peanut breeding. A validated set of PCR Allele Competitive Extension (PACE) markers are now available for MAS of wild species introgressions conferring pathogen resistance. A set of SNP markers were identified for routine mid-density genotyping for GS. The data and tools generated will enable genomics-assisted breeding, which will bolster efficiency in Virginia-type peanut cultivar development. This will result in faster dissemination of elite Virginia-type peanut cultivars to agricultural producers and thereby consumers globally.

## 2 Materials and methods

Methods are provided in brief, complete details and scripts associated with the analyses performed below are provided at https://github.com/USDA-ARS-GBRU/Arachis_cardenasii_Introgression.

### 2.1 Reference genome plant material and sequencing

Virginia-type peanut, *A. hypogaea* subsp. *hypogaea* L. var. *hypogaea* cv. ‘Bailey II’ (**Figure 1**), was used for development of a reference genome sequence. A single Bailey II seed was grown in greenhouse conditions and clonally propagated. At maturity, 68 hour dark treated unexpanded leaves were flash frozen in liquid nitrogen. Nuclei were isolated using the Bionano Prep Plant Tissue DNA Isolation kit (Bionano Genomics, San Diego, CA). Subsequently, high molecular weight (HMW) genomic DNA was extracted for PacBio Continuous Long Read (CLR) sequencing from the isolated nuclei using the Circulomics Nanobind Plant Nuclei Big DNA Kit (Pacific Biosciences, Menlo Park, CA). HMW DNA was sheared with Covaris G-tube (Woburn, MA) to target fragments greater than 20 kb. Sheared DNA was prepared for sequencing using the PacBio SMRTbell Express Template Prep Kit 2.0 (Menlo Park, CA), and size selected with Sage Science’s BluePippin (Beverly, MA). Sequencing was performed on a Sequel II using a 20 hour movie time on 4 SMRT Cells. For optical mapping, 750 ng of ultra-high molecular weight DNA was labeled with the Direct Label and Stain DNA Labeling kit (Bionano Genomics, San Diego, CA) and imaged on a Bionano Saphyr instrument (Bionano Genomics, San Diego, CA)

For short read sequencing, DNA from Bailey II was obtained using the Qiagen DNeasy Plant Kit (Germantown, MD) and evaluated for quality with the Agilent TapeStation (Santa Clara, CA). DNA was sequenced on an Illumina NovaSeq 6000 S2 150 paired-end (PE) flow cell. An additional round of short read sequencing was prepared with the NEBNext Ultra DNA Library Prep Kit for Illumina (Ipswich, MA) and sequenced on a HiSeq 4000 150 PE. Data from both sequencing runs were combined, assessed for quality with FastQC (Andrews, 2010) v. 0.11.9, and cleaned by fastp (Chen et al., 2018) v. 0.20.1.

### 2.2 Transcriptome profiling

Seeds from the Bailey II clones used in the reference genome project were planted at the NCSU Peanut Belt Research Station (Lewiston-Woodville, NC) and maintained with regional agronomic practices. At 126 days after planting, fully expanded mature-leaflets, whole flowers, main and axial stems, pegs, all growth stages of developing pods, and primary and secondary roots were flash-frozen in liquid nitrogen. RNA was extracted using a Sigma-Aldrich Spectrum Plant Total RNA Kit (Saint Louis, MO). Libraries were prepared with the NEBNext Ultra Directional RNA Library Prep Kit for Illumina (Ipswich, MA) and sequenced on an Illumina NovaSeq 6000 S2 platform to generate 150 PE data. Quality assessment and read cleaning was performed in the same manner as for short read DNA.

To generate long-read RNA data, part of the extractions from the fully expanded leaves were used to prepare RNA IsoSeq libraries using standard PacBio protocols. Sequencing was done on a PacBio Sequel II machine.

### 2.3 Genome assembly, annotation, and gene ontology

Bailey II PacBio CLR reads greater than 8,150 bp were assembled by CANU (Koren et al., 2017) v. 1.9. Resulting contigs underwent one round of polishing with Arrow (Pacific BioSciences SMRT Tools Reference Guide, 2019), followed by additional polishing with Pilon (Walker et al., 2014) v. 1.23. Circular contigs, as labeled in the output of CANU (Koren et al., 2017) v. 1.9, were removed from the assembly. Bionano optical data was used to scaffold the assembly and then RagTag (Alonge et al., 2019) was used to generate pseudomolecules. Next, DENTIST (Ludwig et al., 2021) was run to fill gaps between contigs. For quality control, a MUMmer v. 4.0.0beta2 (Marçais et al., 2018) whole genome alignment was performed between the draft Bailey II genome and Tifrunner gnm2 chromosomes. Collapsed regions of the subgenomes were identified, investigated by mapping Bailey II CLR data back to the draft Bailey II genome, and corrected by manually duplicating the collapsed regions to the opposite homeologous chromosome. This formed the final genome sequence, which was deposited to National Center for Biotechnology Information (NCBI) (JAGJTH000000000).

Two RNASeq datasets and one IsoSeq dataset were used to annotate the Bailey II genome through the BIND (Li et al., 2022) annotation pipeline, which was previously tuned for *A. hypogaea* (Campbell & Seetharam, n.d). The first short read RNASeq dataset was from the six Bailey II tissues sequenced here. The second dataset was the *A. hypogaea* gene atlas RNASeq data, which was generated from 22 different tissue types from the Tifrunner cultivar (Clevenger et al., 2016). Bailey II IsoSeq data were collapsed with cupcake (Cupcake, n.d) to obtain a set of full-length, high quality isoforms. These isoforms were incorporated into the BIND pipeline at the Mikado (Venturini et al., 2018) pick step, as the ‘reference’ annotation. Gene Ontologies were assigned to the Bailey II annotation with OmicsBox v. 2.0.36.

### 2.4 WGS panel plant materials and sequencing

A panel of 66 peanut lines that encompass foundational historic lines and lines that represent the phenotypic diversity of the NCSU Virginia-type peanut breeding program, were selected for whole genome sequencing (WGS) to assess the genetic variability present in the breeding program. The panel was composed of 11 historical Virginia-type peanut lines, 14 modern Virginia-type peanut lines, 35 germplasm lines (generally unimproved plant introductions or landraces) - most of which are heavily incorporated into the NCSU breeding population, 2 parental lines, and 4 Runner-type peanut lines (**Table S1**).Young leaf tissue was harvested from a single mature plant per line in the greenhouse. DNA extraction was done with a Qiagen DNeasy Plant Mini Kit (Germantown, MD). Libraries were prepared using the Illumina Truseq Nano DNA prep kit (San Diego, CA) and sequencing was done on an Illumina NovaSeq 6000 platform. WGS data were cleaned by fastp v. 0.20.1 (Chen et al., 2018).

### 2.5 Bailey II reference genome constitution and utility

MUMmer v. 4.0.0beta2 (Marçais et al., 2018) alignment between Bailey II homeologs was conducted to assess similarity between subgenomes. BBTools v. 37.02 basic statistics (Bushnell, n.d), BUSCO v. 4.0.2 (Seppey et al., 2019), and RepeatMasker v. 4.1.2 (Smit et al., 2013) were used to compare genome statistics for Bailey II, Tifrunner gnm1 and 2 (Bertioli et al., 2019), Shitouqi (Zhuang et al., 2019), and Fuhuasheng (Chen et al., 2019) assemblies. MCScanX (Wang et al., 2012) and SynVisio (Bandi & Gutwin, 2020) were run to explore transcriptomic collinearity simultaneously between Bailey II, Tifrunner gnm2, and the diploid progenitor species (Bertioli et al., 2016). LTR Assembly Index (LAI) (Ou et al., 2018) was used to assess genome quality between Bailey II and Tifrunner gnm2. The two reference genomes were further compared to assess which reference allowed for the best alignment of cleaned short read data from all 66 WGS lines sequenced in this study. Unique, concordant read mapping counts were recorded per reference genome, and compared with a Paired Two Sample T-test. Before the test was conducted, two outliers were removed (IL-28 and PI 665000).

### 2.6 WGS panel marker discovery

Cleaned reads were globally aligned against the Bailey II reference genome with Bowtie 2 (Langmead & Salzberg, 2012). SAMtools (Li et al., 2009) was used to remove discordant alignments, remove reads with map quality below 12, and mark optical duplicates. GATK joint calling for variant discovery was conducted in accordance with best practices (Poplin et al., 2018). The resulting set of variant calls were filtered with VCFtools (Danecek et al., 2011) and BCFtools (Danecek et al., 2021) to keep biallelic SNPs with an average read depth between 5 and 200, and to exclude variants on unplaced scaffolds (**Note S1**), sites where the Bailey II short-read sample did not match the Bailey II reference genome, sites that did not have a homozygous alternative genotype (since samples are inbred), and any site with any missing data. The filtered marker set will be called Set 1. Set 1 was then manipulated so that all introgression blocks (**Table S2**) were manually masked, and resulting invariant positions were removed. This masked marker dataset will be referred to as Set 2.

### 2.7 Variant calling in manually duplicated regions of the Bailey II genome

Within regions of the Bailey II genome which were manually duplicated in the genome assembly process, the standard variant calling pipeline described above was modified in the following manner, indicated as lenient variant calling protocol. Within the SAMtools (Li et al., 2009) steps, there was no restriction on mapping quality. Freebayes (Garrison & Marth, 2012) was used for joint calling with no requirements for minimum mapping quality. The resulting set of variant calls were filtered with VCFtools (Danecek et al., 2011) and BCFtools (Danecek et al., 2021) by keeping only biallelic SNPs with an average read depth between 5 and 200, excluding any site with any missing data and removing sites without polymorphism. The remaining markers will be referred to as Set 3.

Two additional variant calling projects were undertaken to ensure that there is biological variation in the manually duplicated regions of the genome. For the first project, CLR data from the Valencia-type peanut cultivar ‘Tennessee Red’ (Bertioli et al., 2022) were aligned against the Bailey II genome by pbmm2 (Li, 2018) and Freebayes (Garrison & Marth, 2012) was used for variant calling. For the second project, a whole genome alignment between Tifrunner gnm2 and Bailey II was conducted with minimap2 (Li, 2018) followed by variant calling with ‘paftools.js’. Variants were matched with sites from marker Set 3 with VCFtools (Danecek et al., 2011) and command line utilities.

### 2.8 Delimit and design markers for *A. cardenasii* introgressions

Nine additional GP-NC WS germplasm lines from the NCSU hexaploid route project were cultivated, sequenced and genotyped with the same protocol as the WGS panel peanut lines (**Note S2**) for the purpose of identifying additional introgression blocks for marker development. Introgression blocks were identified through visualizing genotypes with Genotype Plot (Whiting, 2020/2021) and TASSEL (Bradbury et al., 2007). Within the largest version of each introgression block, sites were selected to design allele-specific assays based on the PACE genotyping chemistry. It was required that all sites be biallelic, not have a SNP within 200 bp of the target site, and have less than 5% missing data. All assays were designed so that the HEX-tagged Y allele corresponded to the *A. cardenasii* allele while the FAM-tagged X allele corresponded to the *A. hypogaea* allele. BatchPrimer3 (You et al., 2008) was used to design all assays using the parameters from Hulse-Kemp et al. (2015). All assays were validated on DNA from individuals known to either contain or lack the introgression in order to test the ability to call the homozygous classes. Pseudo-F_1_ DNA was created by mixing DNA from individuals known to either contain or lack the introgression in order to test the ability to call heterozygotes. PACE assays were run per the manufacturer’s instructions and called with the ‘NCSU PB&G SNP Caller’ (Andres & Dunne, 2022).

### 2.9 Population characterization and selecting SNPs for GS genotyping

To characterize relationships between individuals in the WGS panel, Principal Component Analysis (PCA), population structure, and Identity-By-State (IBS) relationships were computed. PCA of marker Set 1 was done with the SNPRelate (Zheng et al., 2012) R package after a LD prune with a threshold of 0.2. Outliers identified from the first PCA were removed and PCA was rerun. The fastStructure (Raj et al., 2014) program was used to infer population structure from marker Set 2 after the data were randomly thinned by plink v. 1.9 (Chang et al., 2015) to 50,000 variants. SNPRelate was used on marker Set 1 after an LD prune [threshold 0.2] to calculate IBS. The resulting matrix was plotted with the pheatmap (2019) R package. To investigate LD in the WGS panel, haplotype blocks, pairwise r^2^ and LD decay were calculated. Haplotype blocks were calculated in plink v. 1.9 (Chang et al., 2015) with the ‘blocks’ (Taliun et al., 2014) command and a max window of 5 Mb from marker Set 2. Haplotype blocks were visualized with karyoploteR (Gel & Serra, 2017). Markers in Set 1 and 2 were both thinned to one marker per 250 kb and r^2^ between all combinations of markers within a chromosome was determined in plink v. 1.9 (Chang et al., 2015). The pheatmap (2019) package was used to plot the r^2^ relationship matrix. Finally, marker Set 2 was filtered for minor allele frequency < 0.05, randomly thinned to one thousand markers and then used to calculate and plot the LD decay over physical distance with the sommer R package (Covarrubias-Pazaran, 2016; Covarrubias-Pazaran, 2018). Markers from Set 1 and 3 were filtered to generate a high-quality marker set [Set 4] for future use in a mid-density genotyping approach for GS (**Note S3**).

## 3 Results

### 3.1 Bailey II reference genome sequencing and assembly

Sequencing of Virginia-type peanut, ‘Bailey II’, generated greater than 20 million reads of CLR data (NCBI SRR13421161), equating to 93.73x coverage of the estimated 2.7 Gb peanut genome (Samoluk et al., 2015). Cleaned Bailey II short read data totaled 824 million reads, which equated to 41.63x coverage of the peanut genome (NCBI SRR13299519). Cleaned Bailey II RNASeq included more than 3 billion short reads and 34.79 Gb of unique molecular yield (NCBI SRR14146378, SRR14162029-SRR14162033). A total of 225,571 high quality (predicted accuracy ≥ 0.99) and 2,713 low quality (predicted accuracy < 0.99) polished isoforms were obtained (NCBI SRR17412978).

The raw CANU assembly consisted of 1,012 contigs with a total length of 2.54 Gb. Bionano and RagTag (Alonge et al., 2019) scaffolding resulted in 431 scaffolds which represented the 20 chromosomes of *A. hypogaea*. DENTIST (Ludwig et al., 2021) resulted in eleven joins. Comparison of each chromosome of Bailey II to all chromosomes of Tifrunner gnm2 illuminated three distal regions of Bailey II chromosomes that were collapsed into either the A-subgenome or B-subgenome in the assembly process, due to the high sequence similarity between the regions (**Table S3**). The chromosomes with missing information because of the collapse will be referred to as ‘receivers’ as each needed to receive sequence. The remaining chromosomes will be called ‘lenders’ as each had the genetic sequence that needed to be duplicated and added to their respective homeolog. The regions of the lender chromosomes which needed to be duplicated showed an approximate 2-fold change in Bailey II short read depth, when compared to the rest of the lender chromosome. The appropriate lender region was manually duplicated and added onto the receiver to resolve the three collapsed regions in the initial assembly.

The complete Bailey II reference genome consists of 2.56 Gb of sequence in 1,004 contigs (NCBI JAGJTH000000000) (**Table 1**). The A-subgenome has a total sequence of 1.09 Gb while the B-subgenome is larger, with a total sequence of 1.44 Gb. In terms of repetitive content, there were 475,023 detected retroelements and 248,798 detected DNA transposons in the A-subgenome, which equates to 53% and 12% of the sequence total, respectively. In the B-subgenome there were 695,725 detected retroelements and 322,494 detected DNA transposons, which equates to 56% and 12% of the sequence total, respectively. The A-subgenome contained 29,610 annotated genes, whereas the B-subgenome contained 32,444 annotated genes. Chromosome scale information about repetitive and genic content are listed in (**Table S4**). Alignment between homeologs of Bailey II show homeologous pairs 02/12, 04/14, 08/18, and 10/20 are largely collinear, while all others exhibited at least a single inversion.

**Table 1:** Final Bailey II genome assembly statistics as compared to public *A. hypogaea* genomes.

Genome	Bailey II	Fuhuasheng	Shitouqi	Tifrunner gnm1	Tifrunner gnm2
**Scaffold total**	426	29	21	384	442
**Contig total**	1,004	32,721	7,566	4,039	4,139
**Scaffold Length total (bp)**	2,555,804,451	2,551,684,895	2,539,163,406	2,556,916,893	2,557,413,415
**Contig Sequence total (bp)**	2,550,126,081	2,525,211,704	2,538,408,906	2,553,021,534	2,553,632,056
**Scaffold L/N50**	9/136833719	9/137243429	9/135085854	9/135150084	9/135027066
**Contig L/N50**	42/17,573,708	3443/213,557	574/1,293,691	461/1,498,096	464/1,493,114
**Scaffold L/N90**	16/115,852,864	16/111,889,318	16/111,624,253	16/115,504,342	16/116,542,366
**Contig L/N90**	117/6,658,917	10284/74,260	1704/438,763	1461/483,084	1468/482,414
**Max scaffold length (bp)**	159,563,345	168,161,321	159,154,999	160,879,708	160,028,458
**Max contig length (bp)**	61,543,944	1,734,908	8,550,813	9,487,789	9,487,789
**BUSCO Complete**	5,181	5,169	5,179	5,182	5,183
**BUSCO Single Copy**	867	1,156	1,223	863	854
**BUSCO Duplicated**	4,314	4,013	3,956	4,319	4,329
**BUSCO Missing**	168	181	170	169	167
**SINEs**	0.09%	0.09%	0.09%	0.09%	0.09%
**LTR elements**	54.74%	54.81%	54.75%	54.88%	54.88%
**DNA Transposons**	11.47%	11.36%	11.46%	11.48%	11.48%
**Simple Repeats**	1.14%	1.20%	1.07%	1.09%	1.10%
**Low Complexity**	0.35%	0.45%	0.36%	0.39%	0.39%

Benchmarking Universal Single-Copy Orthologs (BUSCO) was run with the same lineage ‘fabales_odb10’ for each genome. The last five rows describe the area that repetitive elements occupy as a percentage of the total genome sequence length. Rows highlighted in blue correspond to contig statistics.

### 3.2 Comparison of genome assemblies

**Figure 2:**
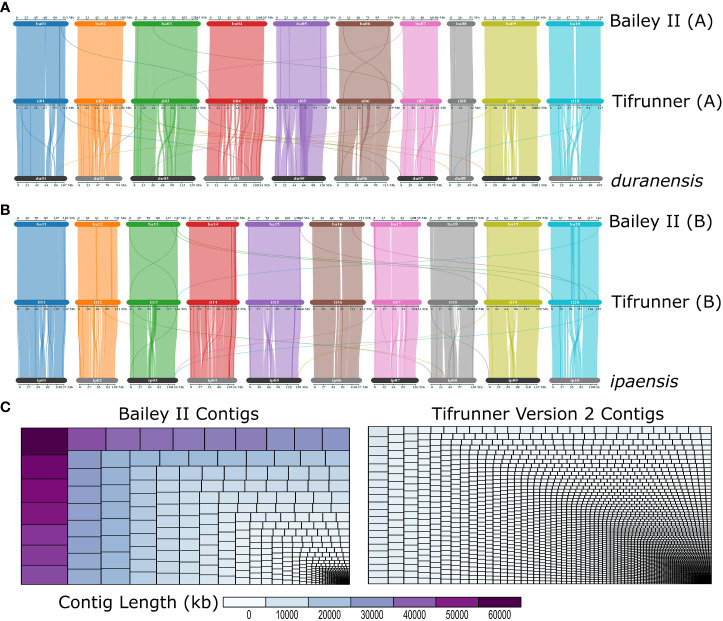
‘Bailey II’ is a high-quality reference peanut genome. **(A)** Three-way comparison of gene organization between the A-subgenome of Bailey II [top], A-subgenome of Tifrunner gnm2 [middle] and *Arachis duranensis* [bottom]. **(B)** Three-way comparison of gene organization between B-subgenome of Bailey II [top], B-subgenome of Tifrunner gnm2 [middle], and *Arachis ipaensis* [bottom]. **(C)** Treemaps depicting Bailey II and Tifrunner gnm2 contig sizes and size distributions. Each rectangle represents a contig that is sized and colored based on contig length in kb. The larger the contig the larger the rectangle.

The Bailey II genome is comparable to publicly available *A. hypogaea* genomes in terms of total sequence length; however, the Bailey II reference genome has greater contiguity than other available peanut reference genomes, as judged by the number of contigs comprising the assemblies. The Bailey II assembly consists of 1,004 contigs, whereas the Tifrunner gnm1, Tifrunner gnm2, Shitouqi and Fuhuasheng reference genomes consist of 4,039, 4,139, 7,566, and 32,721 contigs respectively (**Table 1**; **Figure 2C**). The LAI analysis (**Table S5**) also affirmed the high contiguity of Bailey II. The percentage of genomic content identified as repetitive sequence was similar between all five assemblies compared (**Table 1**). The Bailey II genome had an equivalent BUSCO profile and syntenic transcriptomic organization as the Tifrunner genome (**Table 1**; **Figures 2A, B**). Moreover, both Bailey II and Tifrunner gnm2 had the same trends in transcriptomic collinearity when compared to progenitor chromosomes (**Figures 2A, B**). The unique concordant alignment of WGS data was significantly higher (p-value = 8.987e-16) when Bailey II was used as the reference genome versus Tifrunner gnm2. Data from the WGS population confirmed that Bailey II has *A. cardenasii* introgressions present on Chr02 from 236,159-8,091,659 and Chr08 from 4,010,778-7,096,885 (**Table S2**).

### 3.3 WGS and genome-wide marker discovery

Sequence yield from the full WGS panel was 6.40 billion short reads, which equated to an average coverage of 10.8x per line. Variant discovery identified 1.15 million markers after filtering [marker Set 1] (**Supplementary File 1**). Approximately eight hundred thousand Indels were removed in the filtering process (**Supplementary File 5**). Further filtering was done to remove markers in introgression blocks (**Table S2**), which resulted in 385 thousand remaining markers [marker Set 2] (**Supplementary File 2**). Across the total 21 Mb of manually duplicated assembly regions, the standard variant calling pipeline resulted in zero markers. In those same regions, the lenient variant calling protocol generated a total of 27,310 biallelic variant sites after filters were applied [marker Set 3] (**Supplementary File 3**). Variant calling between Tennessee Red long-read data and Bailey II in the manually duplicated assembly regions confirmed 95 variants which matched those in marker Set 3. Whole genome alignment between Tifrunner gnm2 and Bailey II confirmed 969 variants that matched sites in marker Set 3 (**Table S6**).

### 3.4 Detected *A. cardenasii* introgression blocks and validated PACE assays

**Figure 3:**
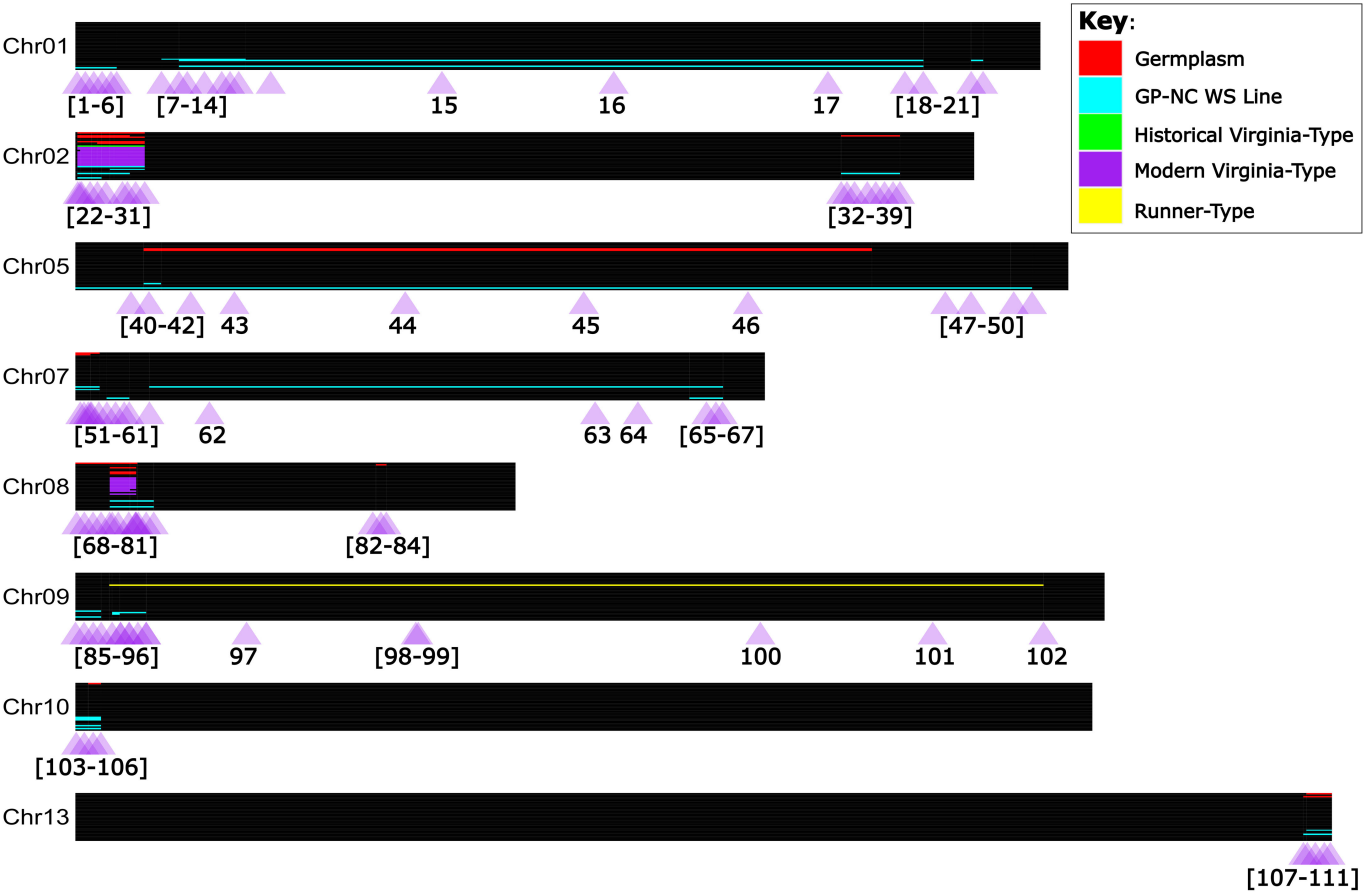
Detected *A. cardenasii* introgression blocks, and validated PCR Allele Competitive Extension marker assays in the context of tetraploid peanut. Chromosomes are represented true to scale as horizontal black bars, with the start of the chromosome at the left hand side (coordinate zero). Individuals are represented as thin rows within the chromosome. *A. cardenasii* blocks are colored by the peanut line type in which the block was detected; purple for modern Virginia-type lines, green for historical Virginia-type lines, yellow for Runner-type lines, blue for ‘GP-NC WS’ lines, and red for germplasm lines. One-hundred and eleven PCR Allele Competitive Extension (PACE) assays were developed for marker assisted selection of *A. cardenasii* blocks, and are represented as purple triangles under each chromosome. The purple triangles are labeled with the assay number which corresponds to **Table S9**.

Of the 75 total lines investigated (the 66 WGS lines plus 9 additional as outlined in Note S2), 36 do not have evidence of *A. cardenasii* in their pedigrees. Of the remaining 39 lines, 34 individuals had a minimum of one introgression block. High marker density allowed for the discrimination of 34 unique introgression blocks on chromosomes 01, 02, 05, 07, 08, 09, 10, and 13 (**Figure 3**; **Table S2**). Sixteen of these unique blocks represent smaller versions of a larger block. Nearly all modern Virginia-type peanut lines sequenced had a pattern of two *A. cardenasii* introgression blocks; 3 Mb in the first quarter of Chr08 and 8 Mb in the first quarter of Chr02. Of all individuals sequenced, only four had the introgression block on Chr13; SPT 10-12, GP-NC WS 2, GP-NC WS 13, and IAC 322. Both SPT 10-12 and GP-NC WS 13 had a short version of the introgression block (143,622,174 - 146,604,204), while IAC 322 and GP-NC WS 2 had a long version of the introgression block (143,264,727 - 146,604,204) (**Figure 3**).

A total of 111 PACE markers were designed and validated for the largest version of each *A. cardenasii* introgression block identified (**Figure 3**; **Table S7**; **Figure S1**). Markers were designed near the limits of the introgression blocks and near known recombination breakpoints [as identified by the WGS data]. For smaller introgressions (<10Mb), assays were placed approximately every 1 Mb within the introgression blocks. For larger introgressions, assays were interspersed throughout, but enriched towards the end of the introgressions.

### 3.5 Population metrics and GS genotyping design

**Figure 4:**
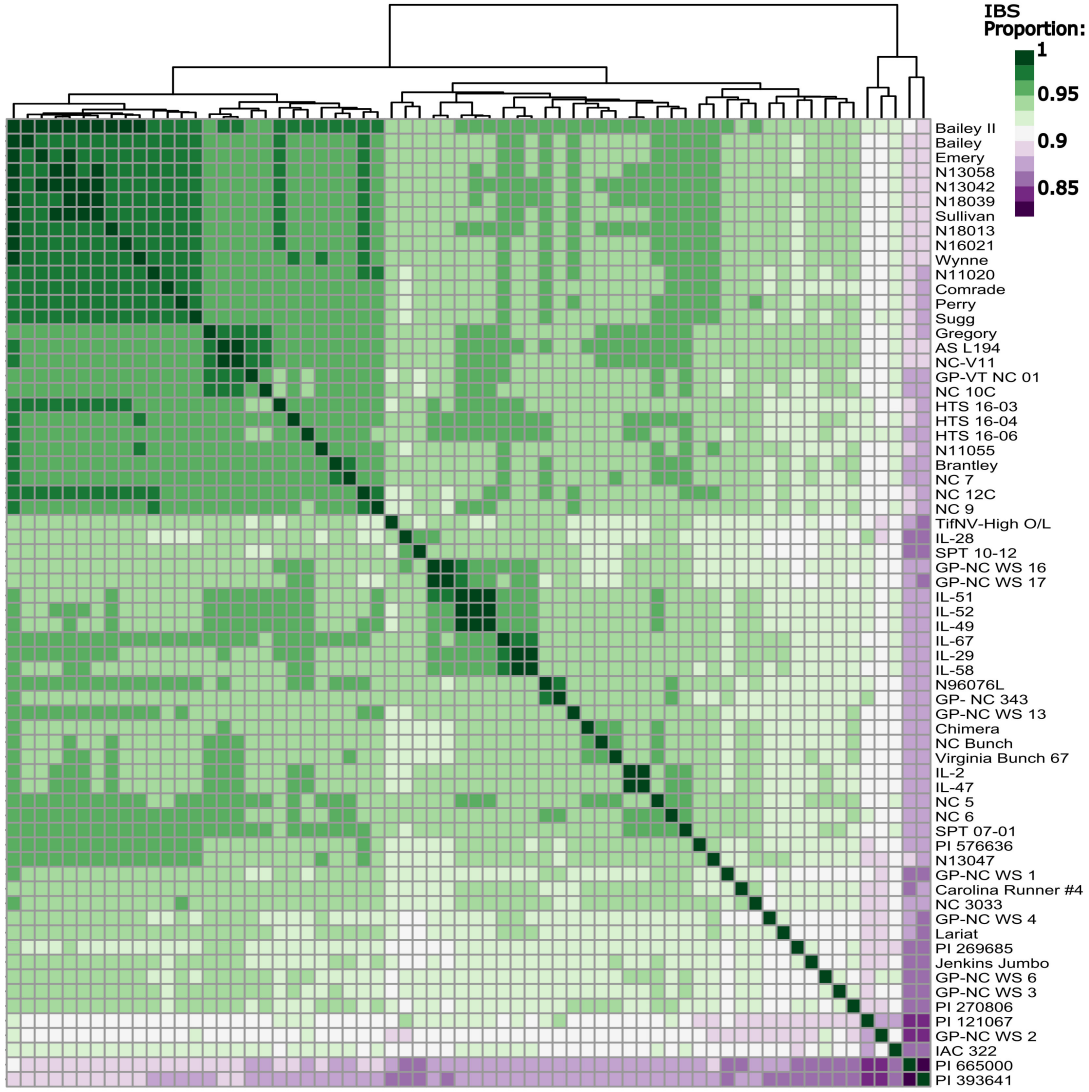
Identity by state matrix of all sixty-six peanut lines included in the whole genome sequencing panel. Dark green represents a higher shared identity, while dark purple represents a lower shared identity. Rows and columns are organized by Euclidean distance.

PCA yielded a very tight cluster of all the WGS lines, besides two extreme outliers, PI 393641 and PI 665000, both of which are germplasm lines. The two outliers were removed, and the PCA was conducted again. This resulted in the majority of the individuals in one tight cluster, with a few new outliers, which were also germplasm lines (**Figure S2**). Looking at IBS between every individual in the population yielded similar results to that of the PCAs (**Figure 4**); the individuals with the lowest degree of IBS were the same germplasm lines that fell out in the PCAs. Modern Virginia-type peanut cultivars and breeding lines had high levels of IBS as expected. FastStructure using marker Set 2 determined there were 4 subpopulations (**Figure S3**).

**Figure 5:**
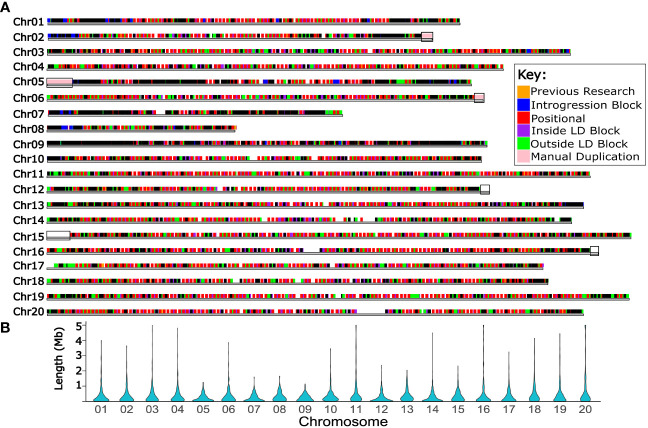
Haplotype blocks informed the selection of forty thousand high quality markers for use in future routine genotyping. **(A)** Twenty chromosomes of Bailey II are represented by horizontal gray bars. Above the chromosomes, marker Set 4 is plotted; each square represents one high quality SNP marker suitable for use in future genomic selection. Colored markers correspond to the primary genomic feature that marker represents (additional information in **Table S8** and **Note S3**). Black boxes overlaid on the chromosomes bars span the regions of the Bailey II genome which had manual duplication. This feature of the genome impacts variant calling and therefore genotyping projects. **(B)** violin plots showing the distribution of haplotype block lengths in Mb for each chromosome.

A total of 4,650 haplotype blocks were identified across the genome, with Chr14 having the most (357) and Chr08 having the least (114) number of haplotype blocks (**Figure 5B**). Comparison of the linkage disequilibrium (r^2^) heat maps generated from marker Sets 1 and 2 show that the introgression blocks create large stretches of the genome in nearly complete linkage disequilibrium (**Figure S4**). Graphical representation of LD decay showed sustained, high levels of LD in the population; where the loess curve never intercepted the standard value of r^2^ = 0.20 (**Figure S5**).

Marker Set 4 consisted of 40,008 high-quality markers that were identified for potential use in a mid-density genotyping approach for GS (**Figure 5A**) (**Supplementary File 4**). **Table S8** lists the number of markers in Set 4 that represent a particular feature of the genome.

## 4 Discussion

### 4.1 Development of *A. cardenasii* PACE assays for MAS

Here we investigate *A. cardenasii* introgression blocks in the context of tetraploid peanut breeding. All lines were genotyped against a tetraploid reference and therefore all introgression blocks are presented in terms of tetraploid genomic coordinates, whereas prior studies have instead utilized comparisons to diploids (Bertioli et al., 2021; Clevenger et al., 2017; Lamon et al., 2020). Contextualizing results in this manner is paramount because advanced peanut cultivar development is done solely at the tetraploid level. Results produced here can now be rapidly consumed by the research community as minimal genomic coordinate manipulation is needed to understand where features lie in cultivated peanuts.

The 34 introgression blocks identified in this study were found across eight chromosomes. The introgression loci across the eight chromosomes are consistent with the previous work of (Bertioli et al., 2021). The blocks identified by Bertioli et al. (2021) collectively covered 418 Mb of the diploid *A. cardenasii* genome [1.13 Gb], which compares favorably with the total of 423 Mb of the tetraploid *A. hypogaea* genome [2.7 Gb] reported here. Our findings are formatted and disseminated as a catalog of unique introgression block boundaries and combinations for use by scientists and breeders (**Figure 3**; **Table S2**). The 111 PACE markers designed, validated, and released in this work (**Figure 3**, **Figure S1**; **Table S7**) can be directly utilized to track the cataloged introgression blocks in *A. hypogaea*. Furthermore, PACE markers can be used in MAS to select for or against specific blocks, detect recombination within blocks, and stack introgression blocks.

The PACE markers released in this work are interspersed within the largest version of each introgression block to allow for recombination within blocks to be detected. Detecting recombination is important in narrowing QTL in order to reduce potential linkage drag of wild alleles. QTL for late leaf spot resistance has been reported from a number of the identified *A. cardenasii* introgression blocks, including the Chr02 first quarter introgression block (Bertioli et al., 2021; Shirasawa et al., 2018; Sujay et al., 2012; Lamon et al., 2020) and from the Chr13 introgression block (Bertioli et al., 2021; Pandey et al., 2017; Sujay et al., 2012; Lamon et al., 2020). Rust and web blotch resistance QTL have been identified from the introgression block on Chr13 (Pandey et al., 2017; Shirasawa et al., 2018; Sujay et al., 2012) and the Chr02 first quarter introgression block (Bertioli et al., 2021) respectively. For nematode resistance, a QTL has been reported on the Chr09 introgression block (Burow et al., 1996; Clevenger et al., 2017; Chu et. al., 2016; Nagy et. al., 2010). Utilizing the marker data in this study to detect recombination, we have identified unique versions of blocks, i.e. individuals containing different amounts of introgressed sequence, for the original introgression blocks. Overall this included 7 unique blocks on the first quarter Chr02 introgression, 4 unique blocks on Chr09, 2 unique blocks on Chr13, and a multitude of uncharacterized introgression blocks on additional chromosomes.

These 34 individuals containing unique blocks, and unique block combinations that are fully genetically characterized in this study, will allow for narrowing QTL in future investigations and offer unique opportunities for breeding. This is especially important for regions under reduced recombination like introgression events which may cause issues such as linkage drag (Zheng et al., 2016). One example may include further study of the root knot nematode resistance locus, which was localized to a 4 Mb segment from the *A. cardenasii* Chr09 introgression block present in ‘RIL 46’ (Chu et al., 2016; Clevenger et al., 2017). Here we determined that GP-NC WS 6 has a similar 4 Mb segment containing the same alleles in the root knot nematode candidate gene region as RIL 46 on the Chr09 introgression block, and it was notably released as a nematode resistant line (Stalker et al., 2002). Moreover, GP-NC WS 2 was found to contain a smaller unique block (less than 1 Mb) nested within the GP-NC WS 6 block. As the GP-NC WS 2 block does not encompass the candidate gene region, it offers a recombination event that would help minimize the search space for true candidate genes (in this case in a negative fashion), as the GP-NC WS 2 line has not been noted as nematode resistant. The blocks on GP-NC WS 2 and 6 show independent support of prior research and demonstrate the utility of unique introgression breakpoints which can be extrapolated for traits associated with other introgression blocks.

WGS data from this study suggests that both the Chr02 first quarter and Chr08 introgression blocks are largely fixed in the modern NCSU Virginia-type peanut breeding population. The PACE markers developed in the present study can be used to rapidly “stack” additional lower frequency introgression blocks atop of the fixed 2-block pattern in the current population. The introgression block on Chr13 would be a good stacking target as it is known to confer multiple disease resistances (Sujay et al., 2012; Pandey et al., 2017; Shirasawa et al., 2018; Lamon et al., 2020; Bertioli et al., 2021), however it is absent from our modern NCSU Virginia-type peanut lines. SPT 10-12 is a valuable germplasm resource for peanut breeders as it carries two QTL introgression blocks. It also has uncharacterized blocks on Chr07, Chr08 and Chr10, and has the ahFAD2B allele for the high-oleic trait, which is now a standard allele for cultivar adoption. Identifying SPT 10-12 as a parent to stack the Chr13 introgression into advanced Virginia-type peanut lines with PACE markers is a direct result of the findings generated in this study.

### 4.2 Bailey II reference genome GS utility

The Bailey II genome is the first Virginia-type peanut reference genome assembled, and it has superior sequence contiguity relative to other public *A. hypogaea* reference genomes. In a GS program, where the genotyping strategy is sequenced-based, the first step in the bioinformatics pipeline is aligning sample data to the reference genome. Characteristics of the reference genome, such as contiguity, accuracy and relationship to the query, influence the success of read alignment. As discussed above, Bailey II has genome statistics that show high contiguity (**Table 1**; **Table S5**); however to assess utility empirically a read mapping study was undertaken. When short reads from all individuals in the WGS panel were aligned, the concordant read alignment was significantly higher using the Bailey II reference genome. This result demonstrates that the Bailey II reference genome has a better performance than Tifrunner gnm2 for samples of interest to the NCSU Virginia-type peanut breeding program. Bailey II is the most appropriate genome to be used for general variant calling and for routine genotyping for GS in the NCSU Virginia-type peanut breeding program.

Additionally, the Bailey II reference genome has less manually duplicated sequence than Tifrunner gnm2. Recent literature suggests peanut should be classified as a segmental allotetraploid due to indication of subgenome exchanges, subgenome conversion, and uniform patterns of homeologous recombination across many cultivated peanut lines (Bertioli et al., 2016; Bertioli et al., 2019; Otyama et al., 2020). High sequence similarity in these regions represents a challenge in genome assembly. First observed in the Tifrunner genome assembly process, all but two of the ten pairs of homeologous chromosomes were affected by collapse of distal regions (Bertioli et al., 2019). To remedy this, regions were copied from one subgenome and manually duplicated onto the corresponding chromosome in the opposite subgenome (Bertioli et al., 2019). In Tifrunner gnm2, 29.9 Mb of the genome is manually duplicated. The Bailey II reference genome has 29.8% less manually duplicated sequence (21.0 Mb) in comparison. Moreover, only three pairs of Bailey II chromosomes are affected rather than eight pairs in Tifrunner, likely due to the improvement in sequencing technology used to develop the different references. The consequence of manual duplication within the genome is the loss of full functionality and interpretation within those regions. For instance, variant calling is affected in regions of manual duplication. During the first stage of variant calling, reads will map equally well to both homeologs causing very poor alignment quality scores. As a ubiquitous bioinformatic quality control measure, reads receiving poor alignment scores are removed. With all the reads removed from those regions, no variant calls will be made, and therefore no genotype data will be available. Historically, progenitor genomes (Bertioli et al., 2016) [or concatenated genomes of the progenitors] were used as the reference in genomic analyses of tetraploid peanut, where genomic manual duplication was not an issue (Peng et al., 2020). As the research community increasingly adopts the tetraploid genome references and sequence-based genotyping technologies, the problem of the manually duplicated genomic regions will heavily affect analyses and interpretations of those analyses. Cognizance of this feature of the Tifrunner and Bailey II genomes is of the utmost importance, as these manually duplicated regions are in telomeric portions of the chromosomes, which are known to contain the highest density of genes (Bertioli et al., 2019).

In our study, lenient variant calling and additional projects showed that there is evidence of variation within those manually duplicated regions (**Table S6**). Future research and sequencing projects are necessary to solve this manual duplication problem. In the interim, it may be best to split the reference genomes into two separate files - one file for the standard genome and one file for one copy of the manually duplicated part of the genome. With limited manually duplicated regions, Bailey II is a suitable reference genome for general variant calling and GS. Awareness of this genomic feature is essential for GS, as markers must be developed and used to represent variation in these regions.

### 4.3 Genome-wide markers for GS

The marker discovery project generated a high volume of informative and well distributed markers. Subsequently, a high-quality subset of those markers were annotated which could be used in a mid-density genotyping system for GS in Runner-type and Virginia-type peanut (*A. hypogaea* subsp. *hypogaea* L. var. *hypogaea*) breeding populations. Both the marker discovery workflow and GS genotyping design done in this work can be scalable and flexible.

The markers presented in this study are informative because they were called from individuals which are representative of the gene pool GS will act upon for the NCSU Virginia-type peanut breeding program. This is especially useful given that nearly all members of the NCSU advanced population have the novel ‘hexaploid route’ introgression event in their pedigree. Detecting 1.15 million markers [Set 1] across the *A. hypogaea* genome is incredible considering markers were determined from just 66 individuals with large degrees of shared ancestry with no missing data allowed. Compared with the preliminary Axiom Arachis2 genotyping project of 200 lines (Hancock, 2018), this marker discovery work represents a 215 fold increase in available markers. In terms of the distribution of marker Set 1, there are approximately 457 markers per 1 Mb. An even and complete distribution of markers across the genome is important to a comprehensive understanding of an individual’s genotype. Of the 1.15 million markers, 66,111 are located within genes as delimited in the Bailey II reference genome annotation, which suggest these markers are particularly informative. The research community may use the bioinformatics pipeline presented in this research to scale up the volume of markers (scripts are available at https://github.com/USDA-ARS-GBRU/Arachis_cardenasii_Introgression/wiki).

Marker Set 4 contains high-quality well distributed SNP markers which can be used to design a genotyping platform such as a SNP array or targeted amplicon-based genotyping system for regular use for GS (**Figure 5A**). Given the estimated target marker count of 3,250 for GS in peanut, the 40,008 markers selected for mid-density genotyping in Set 4 far surpasses the target. Six percent of the markers in Set 4 were specifically placed throughout the genome every 1 Mb to guarantee appropriate distribution. Another six percent of the markers in Set 4 were selected because each are within annotated Bailey II exons, which will enable tracking of variation within these genic regions. The largest share of Set 4, 71%, is dedicated to markers which represent either the haplotype block itself or the recombination hot-spots between neighboring haplotype blocks. Choosing the GS genotyping markers in the context of haplotype blocks led to decreasing the amount of redundant markers, which created space for other markers to be included, such as those in annotated exons. Genotyping using this marker set will be carried out in future work. If using a targeted, amplicon sequencing approach with these markers downstream, the approach will be flexible. Probes can be removed, exchanged, or added over time, which is useful in the context of GS. For example, when an allele becomes fixed in the population, the probe for that allele can be removed or swapped for a probe that targets a new allele. If germplasm is added to the population carrying new alleles, probes can be added to the base set to capture new variation. Overall the markers in this work will enable long-term, flexible genotyping for GS.

### 4.4 GS outlook for the NCSU Virginia-type peanut population

The high resolution genotypic data clarified the relationship between individuals in the WGS population. PCA and IBS analysis shows high genomic similarity between most individuals, barring a few germplasm outliers (**Figure 4**; **Figure S2**). The extremely high level of IBS detected among modern Virginia-type peanut lines meets expectations in that these lines were derived from common parents, and were subjected to the same selection criteria. For population substructure analysis, the optimal number of subpopulations was determined as four; however, visual inspection of subpopulation categories suggests one main population and a few outliers. These results suggest that in future studies, if using advanced Virginia-type peanut lines, minimal population substructure will need to be corrected. From a NCSU Virginia-type peanut breeding perspective, these results suggest a largely homogenous base population. This is beneficial for a long-term recurrent selection program with GS, as drastic gains can be made quickly. After beneficial alleles are fixed through GS, genetic [and inferred phenotypic variation] may be increased through the incorporation of germplasm lines.

LD in the population was measured by r^2^ of pairwise markers (**Figure S4**), which showed that the *A. cardenasii* blocks in the population forces the r^2^ value towards 1 across large segments of the genome. This may pose a challenge for GS in that there is little genetic variability in these large regions. Moreover, it has been previously (Chu et al., 2016; Clevenger et al., 2017) described that recombination within introgression blocks can be rare. Matching previous reports, LD is high, not only in regions of *A. cardenasii* introgressions, but across the entirety of the genome (Hancock, 2018) (**Figure S5**). With large populations and short cycle time, recombination may break up these regions of high LD, which will allow the individuals to be better subjected to selection. Collectively this population-level data will inform the creation of an appropriate training and target population for GS in Virginia-type peanut.

## 5 Conclusion

The objective of this work was to generate the necessary resources, including a reference genome and molecular markers, to initiate MAS and GS for Virginia-type peanut breeding. To establish MAS for *A. cardenasii* introgressions, 34 unique introgression blocks were cataloged in high resolution in the context of tetraploid peanuts. Overall, 111 PACE markers, spanning all introgression blocks cataloged, were designed and validated. These markers can be used to rapidly track, delimit, and stack blocks for future research and breeding purposes. Rust, web blotch, leaf spot and root knot nematode resistances are conferred by *A. cardenasii* blocks contained in germplasm lines sequenced here. PACE markers can be used to introduce favorable blocks from germplasm lines into elite breeding lines to be released as cultivars. The first Virginia-type peanut reference genome was assembled, and it represents large improvements in sequence contiguity and quality. Its utility in short read mapping and reduced amount of manually duplicated sequence it contains, positions ‘Bailey II’ as a prominent reference genome for routine variant calling and use in GS. The sequencing of a panel of 66 important peanut lines to the Virginia-type peanut breeding program allowed for the capture of 1.15 million informative variant sites across the *A. hypogaea* genome. A subset of 40,008 high-quality markers were identified for use in a mid-density genotyping system for future GS. The high marker density also led to determining that most individuals in the WGS panel had a very close genomic relationship. This suggests a homogeneous population of advanced breeding materials fit for GS. With the findings of this study, applied genomics-informed breeding may commence in the NCSU Virginia-type peanut breeding program.

## Data availability statement

The data presented in the study are deposited in the NCBI BioProject PRJNA796025 and BioProject PRJNA675616. Accession numbers can be found in the **Supplementary Material**. All scripts for this study can be found in the GitHub Repository https://github.com/USDA-ARS-GBRU/Arachis_cardenasii_Introgression/wiki.

## Author contributions

CN - First Author; Data Curation; Formal Analysis; Investigation; Methodology; Visualization; and Writing. RA - Second Author; Conceptualization; Project administration; Resources; Funding Acquisition; Supervision; Data Analysis; and Writing - Editing & Review. RY - Investigation; Genome Sequencing Resources; Methodology; and Validation. JC - Investigation; Genome Annotation Resources; Methodology; and Validation. SS - Project Supervision; Resources; and Writing - Editing & Review. SC - Project Supervision; Sequencing Methodology; Resources; and Writing - Editing & Review. BS - Project Administration; Sequencing Resources; Methodology; Data Curation; Supervision; Writing - Editing & Review. AO - Investigation; Methodology; Supervision. AH-K - Corresponding Author; Project Administration; Investigation; Resources; Methodology; Funding Acquisition; Supervision; Writing - Editing & Review. JD- Corresponding Author; Project Administration; Conceptualization; Investigation; Resources; Methodology; Funding Acquisition; Supervision; Writing - Editing & Review. All authors contributed to the article and approved the submitted version.
