# Supplementary material for: Initiation of genomics-assisted breeding in Virginia-type peanuts through the generation of a de novo reference genome and informative markers
Source: Front Plant Sci. 2023 Jan 27;13:1073542. doi: 10.3389/fpls.2022.1073542 (PMC9911918; doi:10.3389/fpls.2022.1073542)
Supplement: Supplementary file 1 [file DataSheet_1.docx]

**Supplementary**

**Table S1.** Peanut Lines for Whole Genome Sequencing categorized by line type. Lines categorized as ‘Runner-Type’, ‘Modern Virginia-Type’ and ‘Historical Virginia-Type’ are classified as *Arachis hypogaea* subsp. *hypogaea* L. var. *hypogaea*. Germplasm and Parental lines may be in the subspecies *fastigiata*, *hypogaea,* or represent an intermediate classification. All sequences are deposited in the National Center for Biotechnology Information under project PRJNA796025.

| **Line Name** | **Line Type** | **Sequence Read Archive ID** |
| --- | --- | --- |
| AS L194 | Runner-Type | SRR17652172 |
| Bailey | Modern Virginia-Type | SRR17652166 |
| Bailey II | Modern Virginia-Type | SRR17652155 |
| Brantley | Historical Virginia-Type | SRR17652141 |
| Carolina Runner #4 | Germplasm | SRR17652199 |
| Chimera | Germplasm | SRR17652108 |
| Comrade | Modern Virginia-Type | SRR17652110 |
| Emery | Modern Virginia-Type | SRR17652144 |
| GP- NC 343 | Germplasm | SRR17652175 |
| GP-NC WS 1 | Germplasm | SRR17652111 |
| GP-NC WS 13 | Germplasm | SRR17652189 |
| GP-NC WS 16 (SPT 06-06) | Germplasm | SRR17652185 |
| GP-NC WS 17 (SPT 06-07) | Germplasm | SRR17652150 |
| GP-NC WS 2 | Germplasm | SRR17652198 |
| GP-NC WS 3 | Germplasm | SRR17652197 |
| GP-NC WS 4 | Germplasm | SRR17652196 |
| GP-NC WS 6 | Germplasm | SRR17652194 |
| GP-VT NC 01 | Germplasm | SRR17652168 |
| Gregory | Historical Virginia-Type | SRR17652165 |
| HTS 16-03 | Germplasm | SRR17652118 |
| HTS 16-04 | Germplasm | SRR17652117 |
| HTS 16-06 | Germplasm | SRR17652116 |
| IL-2 | Germplasm | SRR17652149 |
| IL-28 | Germplasm | SRR17652181 |
| IL-29 | Germplasm | SRR17652180 |
| IL-47 | Germplasm | SRR17652115 |
| IL-49 | Germplasm | SRR17652106 |
| IL-51 | Germplasm | SRR17652179 |
| IL-52 | Germplasm | SRR17652109 |
| IL-58 | Germplasm | SRR17652178 |
| IL-67 | Germplasm | SRR17652105 |
| IAC 322 | Germplasm | SRR17652169 |
| Jenkins Jumbo | Parental | SRR17652146 |
| Lariat | Runner-Type | SRR17652113 |
| N11020 | Modern Virginia-Type | SRR17652114 |
| N11055 | Modern Virginia-Type | SRR17652137 |
| N13042 | Modern Virginia-Type | SRR17652138 |
| N13047 | Modern Virginia-Type | SRR17652120 |
| N13058 | Modern Virginia-Type | SRR17652142 |
| N16021 | Modern Virginia-Type | SRR17652176 |
| N18013 | Modern Virginia-Type | SRR17652192 |
| N18039 | Modern Virginia-Type | SRR17652135 |
| N96076L | Germplasm | SRR17652182 |
| NC 10C | Historical Virginia-Type | SRR17652124 |
| NC 12C | Historical Virginia-Type | SRR17652140 |
| NC 3033 | Germplasm | SRR17652143 |
| NC 5 | Historical Virginia-Type | SRR17652164 |
| NC 6 | Historical Virginia-Type | SRR17652163 |
| NC 7 | Historical Virginia-Type | SRR17652139 |
| NC 9 | Historical Virginia-Type | SRR17652125 |
| NC Bunch | Parental | SRR17652121 |
| NC-V11 | Historical Virginia-Type | SRR17652119 |
| Perry | Historical Virginia-Type | SRR17652162 |
| PI 121067 | Germplasm | SRR17652173, SRR17652174 |
| PI 269685 | Germplasm | SRR17652184, SRR17652183 |
| PI 270806 | Germplasm | SRR17652171 |
| PI 393641 | Germplasm | SRR17652123 |
| PI 576636 | Germplasm | SRR17652170 |
| PI 665000 | Germplasm | SRR17652167 |
| SPT 07-01 | Germplasm | SRR17652128 |
| SPT 10-12 | Germplasm | SRR17652127 |
| Sugg | Historical Virginia-Type | SRR17652161 |
| Sullivan | Modern Virginia-Type | SRR17652133 |
| TifNV-High O/L | Runner-Type | SRR17652157 |
| Virginia Bunch 67 | Runner-Type | SRR17652107 |
| Wynne | Modern Virginia-Type | SRR17652122 |

**Table S2.** Coordinates for *A. cardenasii* blocks within *A. hypogaea* lines detected in this study. Coordinates are relative to the Bailey II reference genome. Lines which are homozygous or heterozygous for the blocks are included.

| **Chromosome** | **Start Coordinate (Mb)** | **End Coordinate (Mb)** | **Samples with Block** |
| --- | --- | --- | --- |
| 1 | 0.000 | 4.823 | GP-NC WS 9 |
| 1 | 4.823 | 10.039 | NONE |
| 1 | 10.039 | 12.085 | GP-NC WS 13 |
| 1 | 12.085 | 19.870 | GP-NC WS 13, GP-NC WS 1, GP-NC WS 11 |
| 1 | 19.870 | 98.952 | GP-NC WS 1, GP-NC WS 11 |
| 1 | 98.952 | 104.499 | NONE |
| 1 | 104.499 | 105.917 | GP-NC WS 1 |
| 1 | 105.917 | 112.592 | NONE |
| 2 | 0.000 | 0.236 | NONE |
| 2 | 0.236 | 0.560 | Bailey, Bailey II, N11020, GP-NC WS 2, GP-NC WS 3, GP-NC WS 6, HTS 16-04, IAC 322, N11055, N13042, N13047, N13058, N16021, N18013, N18039, N96076L, SPT 10-12, Sugg, Sullivan, Wynne, GP-NC WS 9 |
| 2 | 0.560 | 0.695 | Emery, Bailey, Bailey II, N11020, GP-NC WS 2, GP-NC WS 3, GP-NC WS 6, HTS 16-04, IAC 322, N11055, N13042, N13047, N13058, N16021, N18013, N18039, N96076L, SPT 10-12, Sugg, Sullivan, Wynne, GP-NC WS 9 |
| 2 | 0.695 | 1.890 | Emery, Bailey, Bailey II, N11020, GP-NC WS 2, GP-NC WS 3, HTS 16-04, IAC 322, N11055, N13042, N13047, N13058, N16021, N18013, N18039, N96076L, SPT 10-12, Sugg, Sullivan, Wynne, GP-NC WS 9 |
| 2 | 1.890 | 2.548 | Emery, Bailey, Bailey II, N11020, GP-NC WS 2, GP-NC WS 3, HTS 16-04, IAC 322, N11055, N13042, N13047, N13058, N16021, N18013, N18039, N96076L, SPT 10-12, Sugg, Sullivan, Wynne, GP-NC WS 9 |
| 2 | 2.548 | 3.052 | HTS 16-06, Emery, Bailey, Bailey II, N11020, GP-NC WS 2, GP-NC WS 3, HTS 16-04, IAC 322, N11055, N13042, N13047, N13058, N16021, N18013, N18039, N96076L, SPT 10-12, Sugg, Sullivan, Wynne, GP-NC WS 9 |
| 2 | 3.052 | 4.028 | HTS 16-06, Emery, Bailey, Bailey II, N11020, GP-NC WS 2, GP-NC WS 3, HTS 16-04, IAC 322, N11055, N13042, N13047, N13058, N16021, N18013, N18039, N96076L, SPT 10-12, Sugg, Sullivan, Wynne |
| 2 | 4.028 | 4.519 | HTS 16-06, Emery, Bailey, Bailey II, N11020, GP-NC WS 2, GP-NC WS 3, HTS 16-04, IAC 322, N11055, N13042, N13047, N13058, N16021, N18013, N18039, N96076L, SPT 10-12, Sugg, Sullivan, Wynne, GP-NC WS 13 |
| 2 | 4.519 | 6.354 | HTS 16-06, Emery, Bailey, Bailey II, N11020, GP-NC WS 2, GP-NC WS 3, HTS 16-04, IAC 322, N11055, N13042, N13047, N13058, N16021, N18013, N18039, N96076L, SPT 10-12, Sugg, Sullivan, Wynne, GP-NC WS 13 |
| 2 | 6.354 | 8.091 | HTS 16-06, Emery, Bailey, Bailey II, N11020, GP-NC WS 3, HTS 16-04, N11055, N13042, N13047, N13058, N16021, N18013, N18039, N96076L, SPT 10-12, Sugg, Sullivan, Wynne ,GP-NC WS 13 |
| 2 | 8.091 | 89.331 | NONE |
| 2 | 89.331 | 96.210 | GP-NC WS 2, IAC 322 |
| 2 | 96.210 | 104.866 | NONE |
| 3 | 0 | 142.931 | NONE |
| 4 | 0 | 124.642 | NONE |
| 5 | 0 | 7.965 | GP-NC WS 9 |
| 5 | 7.965 | 10.000 | GP-NC WS 9, GP-NC WS 2, GP-NC WS 6, GP-NC WS 12, HTS IL-2, HTS IL-47 |
| 5 | 10.000 | 92.936 | GP-NC WS 9, GP-NC WS 6, GP-NC WS 12, HTS IL-2, HTS IL-47 |
| 5 | 92.936 | 109.132 | GP-NC WS 9, GP-NC WS 6 |
| 5 | 109.132 | 111.625 | GP-NC WS 9 |
| 5 | 111.625 | 115.852 | NONE |
| 6 | 0 | 119.095 | NONE |
| 7 | 0 | 1.754 | SPT 07-01, SPT 10-12, GP-NC WS 3, GP-NC WS 13 |
| 7 | 1.754 | 2.832 | SPT 10-12, GP-NC WS 3, GP-NC WS 13 |
| 7 | 2.832 | 3.639 | NONE |
| 7 | 3.639 | 6.303 | GP-NC WS 9 |
| 7 | 6.303 | 8.613 | NONE |
| 7 | 8.613 | 71.642 | GP-NC WS 3 |
| 7 | 71.642 | 75.569 | GP-NC W 3, GP-NC WS 9 |
| 7 | 75.569 | 80.447 | NONE |
| 8 | 0 | 4.010 | SPT 10-12 |
| 8 | 4.010 | 6.365 | SPT 10-12, Wynne, Sullivan, N13042, N13058, N16021, N18013, N18039, N96076L, HTS 16-04, HTS 16-06, GP-NC WS 1, GP-NC WS 11, Emery, N11020, Bailey, Bailey II |
| 8 | 6.365 | 7.096 | SPT 10-12, Wynne, Sullivan, N13042, N13058, N18013, N18039, N96076L, HTS 16-04, HTS 16-06, GP-NC WS 1, GP-NC WS11, Emery, N11020, Bailey, Bailey II |
| 8 | 7.096 | 7.240 | GP-NC WS 1, GP-NC WS 11, SPT 10-12 |
| 8 | 7.240 | 9.149 | GP-NC WS 1, GP-NC WS 11 |
| 8 | 9.149 | 35.070 | NONE |
| 8 | 35.070 | 36.300 | SPT 07-01, GP-NC WS 15, GP-NC WS 12 |
| 8 | 36.300 | 51.353 | NONE |
| 9 | 0 | 3.008 | GP-NC WS 1, GP-NC WS 11 |
| 9 | 3.008 | 3.958 | NONE |
| 9 | 3.958 | 4.286 | TifNV-High O/L |
| 9 | 4.286 | 5.176 | TifNV-High O/L, GP-NC WS 2, GP-NC WS 6 |
| 9 | 5.176 | 8.267 | TifNV-High O/L, GP-NC WS 6 |
| 9 | 8.267 | 112.959 | TifNV-High O/L |
| 9 | 112.959 | 120.081 | NONE |
| 10 | 0 | 1.497 | GP-NC WS 13, GP-NC WS 3, GP-NC WS 4, GP-NC WS 8, GP-NC WS 9 |
| 10 | 1.497 | 2.991 | GP-NC WS 13, GP-NC WS 3, GP-NC WS 4, GP-NC WS 8, GP-NC WS 9, SPT 10-12 |
| 10 | 2.991 | 118.652 | NONE |
| 11 | 0 | 148.420 | NONE |
| 12 | 0 | 120.875 | NONE |
| 13 | 0 | 143.264 | NONE |
| 13 | 143.264 | 143.622 | GP-NC WS 2, IAC 322 |
| 13 | 143.622 | 146.604 | GP-NC WS 2, IAC 322, GP-NC WS 13, SPT 10-12 |
| 14 | 0 | 143.336 | NONE |
| 15 | 0 | 159.563 | NONE |
| 16 | 0 | 150.585 | NONE |
| 17 | 0 | 135.507 | NONE |
| 18 | 0 | 136.833 | NONE |
| 19 | 0 | 159.046 | NONE |
| 20 | 0 | 146.426 | NONE |

**Table S3.**  Regions and directions of collapse in the Bailey II genome assembly process. For the location of collapse, ‘beginning’ is the end of the chromosome with coordinate 0, and ‘end’ is the part of the chromosome farthest from the 0 coordinate. The lender chromosome’s sequence was copied and added to the receiver chromosome in each case.

| Homeologous Chromosomes Involved | Direction of Collapse | Location of Collapse | Lender  Chromosome | Receiver Chromosome | Length DNA Duplicated and Added to the Receiver (bp) |
| --- | --- | --- | --- | --- | --- |
| 02/12 | A-subgenome | end | 02 | 12 | 2,241,792 |
| 05/15 | B-subgenome | beginning | 15 | 05 | 6,377,890 |
| 06/16 | B-subgenome | end | 16 | 06 | 1,885,323 |

**Table S4.** Genes and repetitive elements per chromosome of the Bailey II reference genome

| **Chromosome Name** | **Length (bp)** | **Annotated Genes (Count)** | **Retroelements (Percent Sequence Length)** | **DNA transposons (Percent Length)** |
| --- | --- | --- | --- | --- |
| arahy.BaileyII.gnm1.chr01 | 112592960 | 2918 | 54% | 11% |
| arahy.BaileyII.gnm1.chr02 | 104866288 | 2725 | 53% | 12% |
| arahy.BaileyII.gnm1.chr03 | 142931864 | 4217 | 53% | 12% |
| arahy.BaileyII.gnm1.chr04 | 124642178 | 2691 | 59% | 11% |
| arahy.BaileyII.gnm1.chr05 | 115852864 | 3274 | 53% | 11% |
| arahy.BaileyII.gnm1.chr06 | 119095274 | 3077 | 55% | 11% |
| arahy.BaileyII.gnm1.chr07 | 80447606 | 2154 | 52% | 12% |
| arahy.BaileyII.gnm1.chr08 | 51353827 | 2814 | 29% | 12% |
| arahy.BaileyII.gnm1.chr09 | 120081157 | 2979 | 56% | 12% |
| arahy.BaileyII.gnm1.chr10 | 118652483 | 2761 | 57% | 11% |
| arahy.BaileyII.gnm1.chr11 | 148420878 | 2907 | 59% | 12% |
| arahy.BaileyII.gnm1.chr12 | 120875274 | 2863 | 53% | 12% |
| arahy.BaileyII.gnm1.chr13 | 146604204 | 4444 | 51% | 12% |
| arahy.BaileyII.gnm1.chr14 | 143336434 | 3162 | 57% | 12% |
| arahy.BaileyII.gnm1.chr15 | 159563345 | 3376 | 58% | 11% |
| arahy.BaileyII.gnm1.chr16 | 150585916 | 3255 | 57% | 12% |
| arahy.BaileyII.gnm1.chr17 | 135507158 | 2933 | 57% | 11% |
| arahy.BaileyII.gnm1.chr18 | 136833719 | 2862 | 56% | 11% |
| arahy.BaileyII.gnm1.chr19 | 159046102 | 3391 | 57% | 11% |
| arahy.BaileyII.gnm1.chr20 | 146426242 | 3251 | 58% | 12% |
| SUBGENOME A | 1090516501 | 29610 | 53% | 12% |
| SUBGENOME B | 1447199272 | 32444 | 56% | 12% |

**Table S5.** Inspecting genome quality with the Long Terminal Repeat Assembly Index (LAI) for Bailey II and Tifrunner version two per subgenome.

| **Organism** | **Subgenome** | **Raw LAI** | **LAI** |
| --- | --- | --- | --- |
| Bailey II | A | 21.61 | 23.65 |
| Tifrunner | A | 20.79 | 22.13 |
| Bailey II | B | 19.9 | 20.1 |
| Tifrunner | B | 19.4 | 19.02 |

**Table S6.** Variant calling on the computationally duplicated regions of the Bailey II genome. Column one describes the regions of Bailey II genome which have been computationally duplicated, column two shows the length of the region, column three has the count of markers from marker Set 1 in that region, column four has the marker count for marker Set 3 in that region, column five shows how many variants from the PacBio Tennessee Red data match with marker Set 3, and column six shows how many variants from the Tifrunner gnm2 whole genome alignment match the sites in marker Set 3.

| **Genomic Region (chr<#>:<start>..<end>)** | **Length of Region (bp)** | **Marker Set 1 (marker count)** | **Marker Set 3 (marker count)** | **Tennessee Red Long Read Supported (marker count)** | **Tifrunner gnm2 Whole Genome Alignment Supported (marker count)** |
| --- | --- | --- | --- | --- | --- |
| chr02:102624496..104866288 | 2,241,792 | 0 | 2866 | 2 | 126 |
| chr12:118633482..120875274 | 2,241,792 | 0 | 2904 | 2 | 123 |
| Chr05: 0..6377890 | 6,377,890 | 0 | 8073 | 50 | 272 |
| Chr15: 0..6377890 | 6,377,890 | 0 | 7819 | 41 | 279 |
| Chr06: 117209951..119095274 | 1,885,323 | 0 | 2890 | 0 | 84 |
| Chr16: 148700593..150585916 | 1,885,323 | 0 | 2758 | 0 | 85 |

**Table S7.** PACE Assays for use in MAS for *A. cardenasii* blocks

<SEPARATE FILE>

**Table S8.** High-Quality markers selected for future use in mid-density genotyping for genomic selection; identified as marker Set 4. The 40,008 markers were selected given their membership in at least one category (category ID in the table corresponds to Note S3). Counts/percentages in the table do not sum to 40,008/100% because markers may have membership in more than one category.

| **ID** | **Category** | **Marker Count** | **Percentage of Total Markers Selected** |
| --- | --- | --- | --- |
| A | Position matches an informative marker from previous research | 293 | 0.73% |
| B | Markers within *A. cardenasii* introgression blocks | 1,128 | 2.83% |
| C | Markers representing A) haplotype blocks and B) recombination hotspots between haplotype blocks | 28,506 | 71.27% |
| D | Markers spaced every 1 Mb | 2,326 | 5.82% |
| E | Markers from Set 3 - representing possible variation within manually duplicated parts of the Bailey II genome | 6,915 | 17.06% |
| F | Markers within annotated exons | 2,200 | 5.50% |
|  | **TOTAL** | **40,008** | **100%** |

FAM

**C.**

**D.**


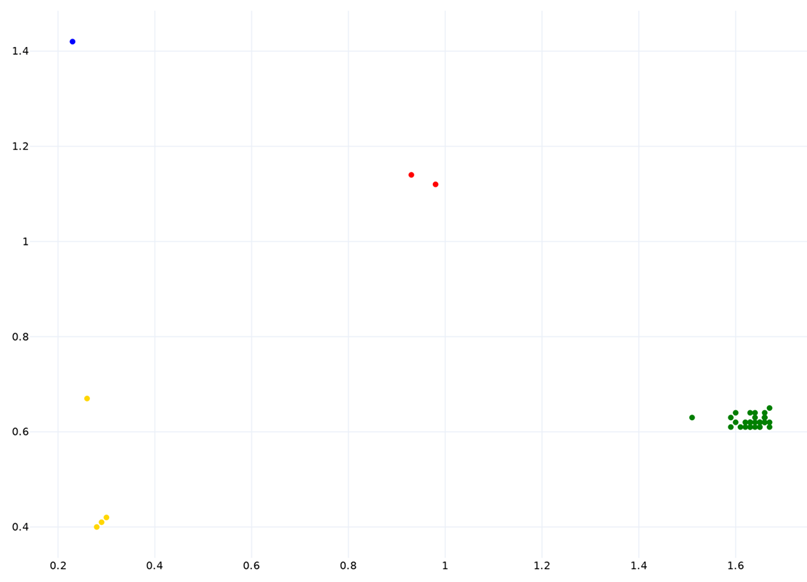

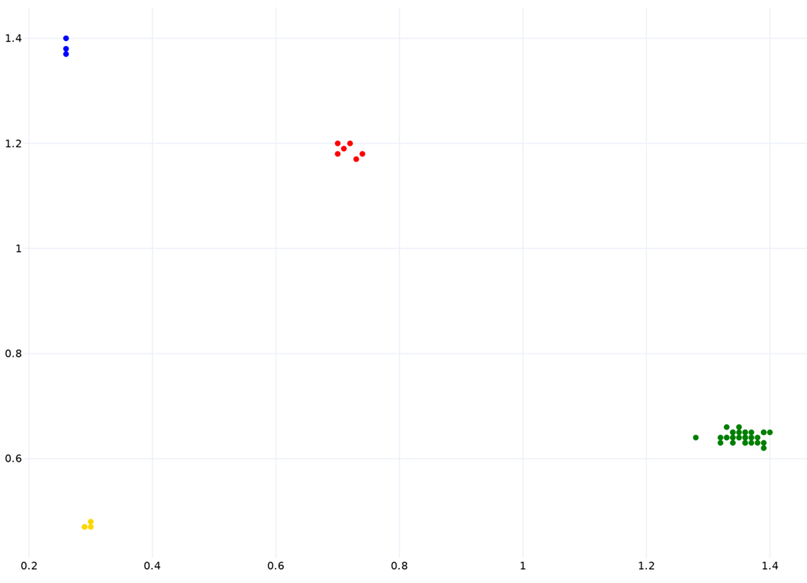


**Assay 107, Chr13_143,276,714**

**Assay 111, Chr13_146,423,518**

HEX

**A.**


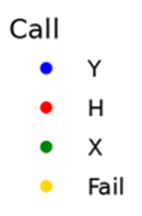

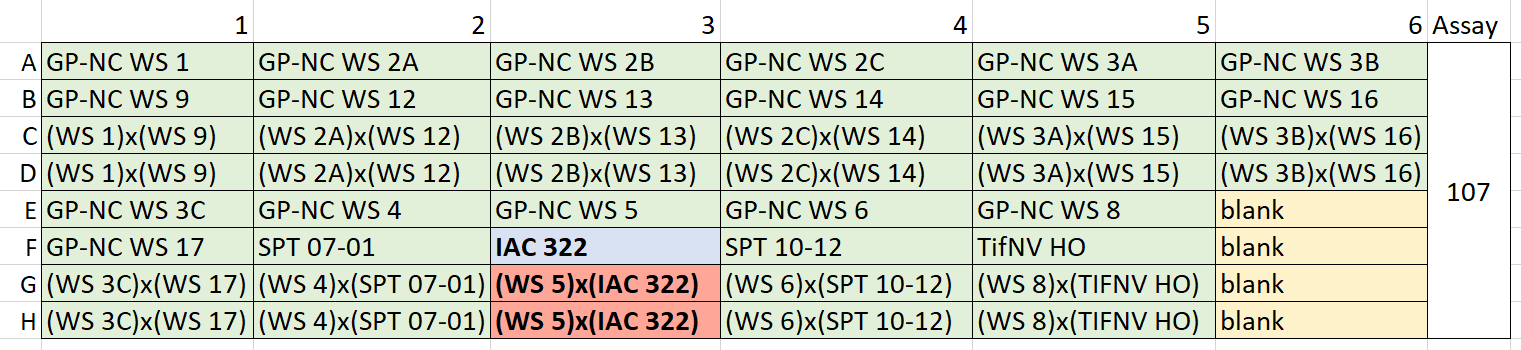

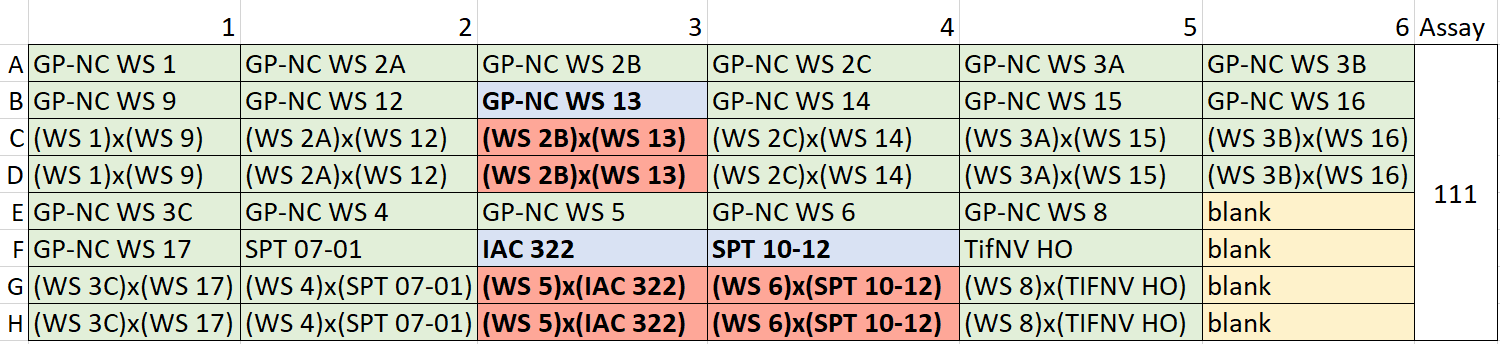


**B.**

**Figure S1.** PCR Allele Competitive Extension assay validation for assays 107 and 111, which were designed for the chromosome 13 *A. cardenasii* introgression blocks. A and B) Scatterplots for assay 107 and 111 respectively, where blue points represent the homozygous *A. cardenasii* genotype, red points represent the heterozygous genotype, green points represent the homozygous *A. hypogaea* genotype and yellow points are negative non-template controls. The ‘NCSU PB&G SNP Caller’ (snp-caller.herokuapp.com) was used to call the genotypes and create the scatterplots presented here. C and D) Plate layouts corresponding to the individuals genotyped and plotted in the scatterplots for assays 107 and 111 respectively. The individuals are colored by their called genotype. Notice that IAC 322 has a longer version of the *A. cardenasii* block on Chromosome 13, then SPT 10-12 and GP-NC WS 13 do, which is supported by the whole genome sequencing data.


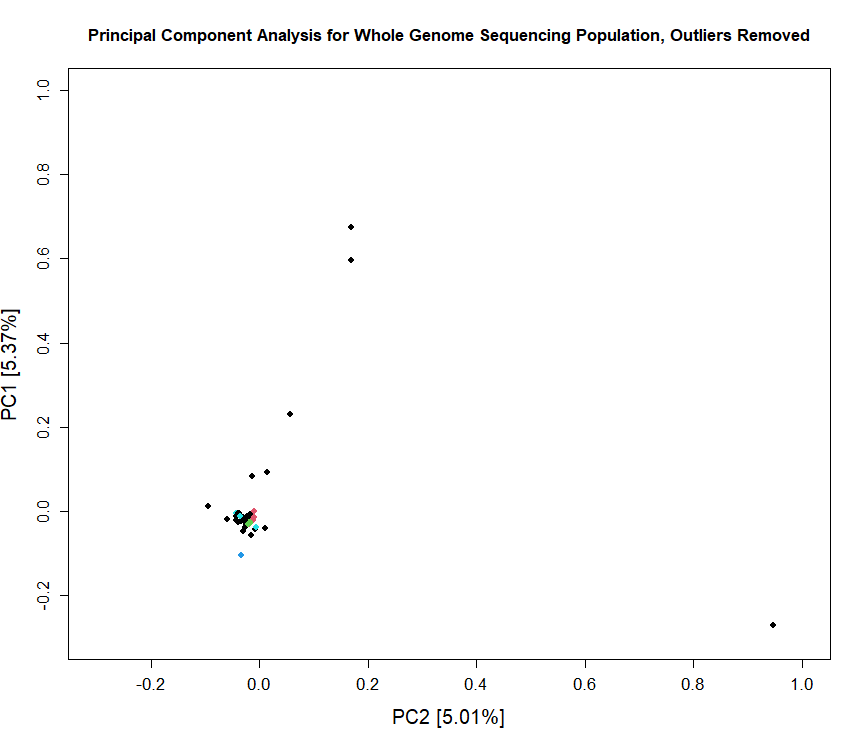


GP-NC WS 2

GP-NC WS 6

IAC 322

PI 269685

GP-NC WS 3

GP-NC WS 4

PI 121067

Jenkins Jumbo


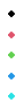


Germplasm

Historical Virginia-Type

Modern Virginia-Type

Parental

Runner

**Figure S2.** Principal Component Analysis of the peanut lines used for whole genome sequencing. Individuals PI 393641 and PI 665000 were removed as they were determined to be outliers.


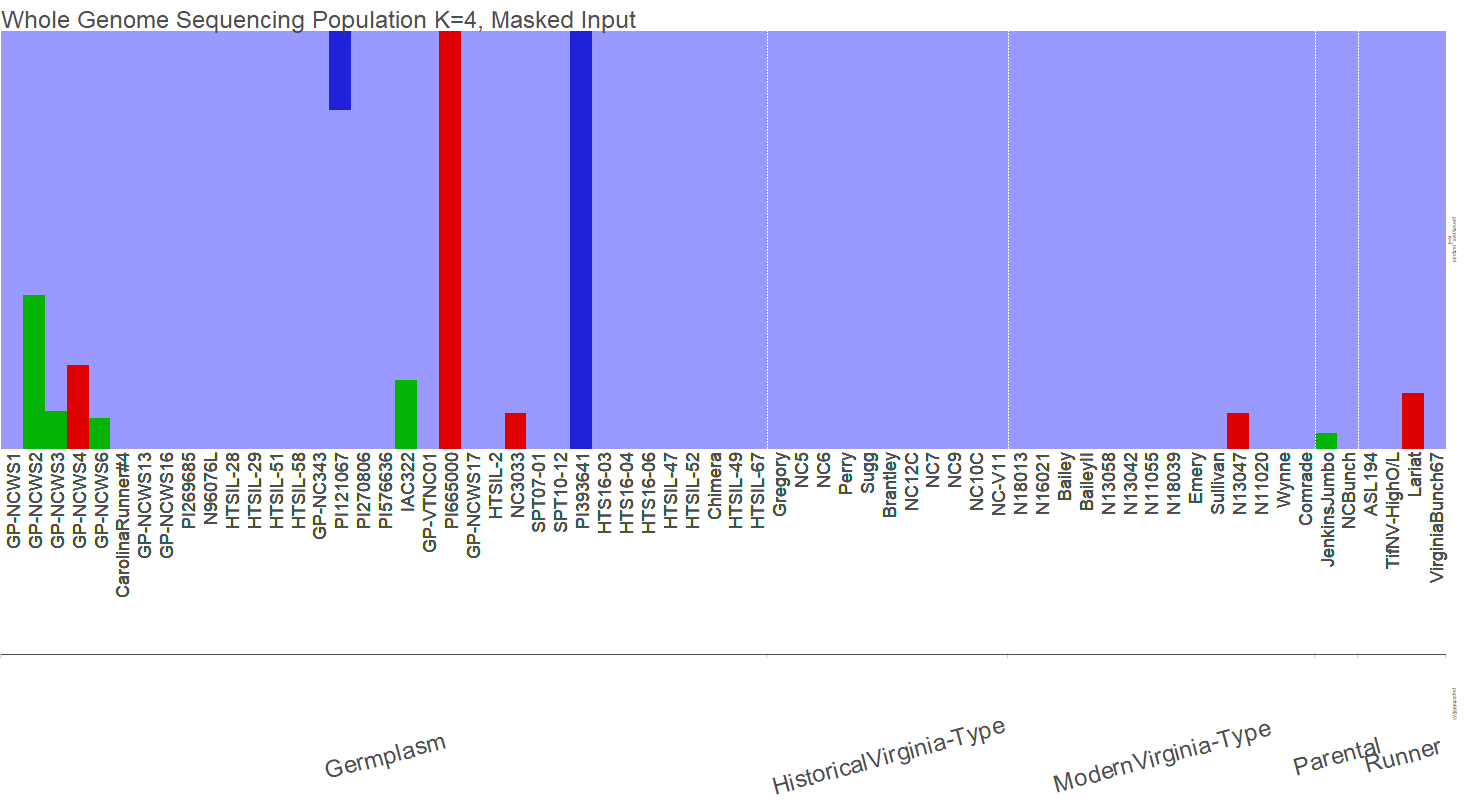


**Germplasm**

**Historical Virginia-Type**

**Modern Virginia-Type**

**Parental**

**Runner**

**-Type**

**Figure S3**. Graphical representation of population structure (k=4) in the whole genome sequencing population.


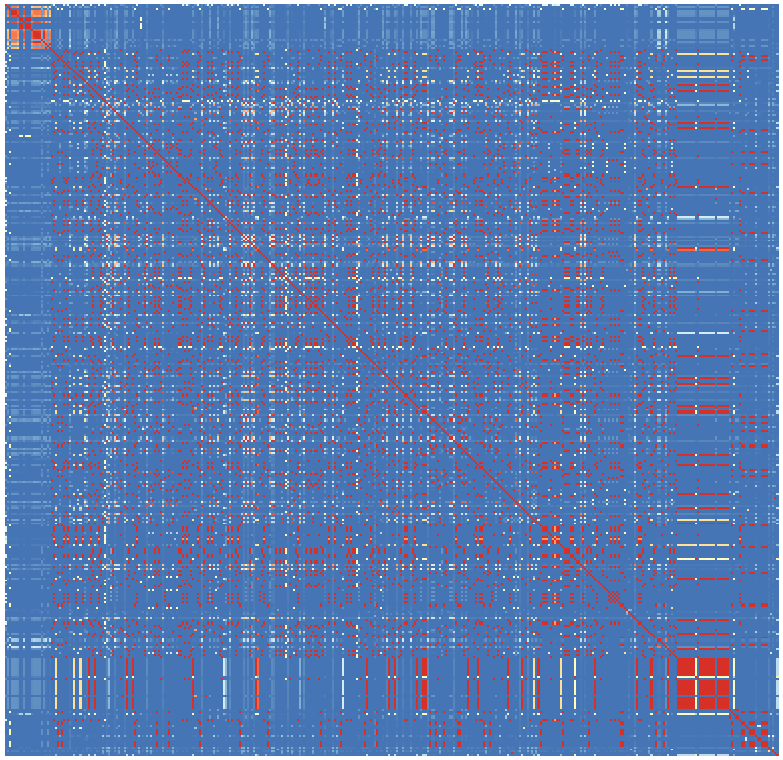

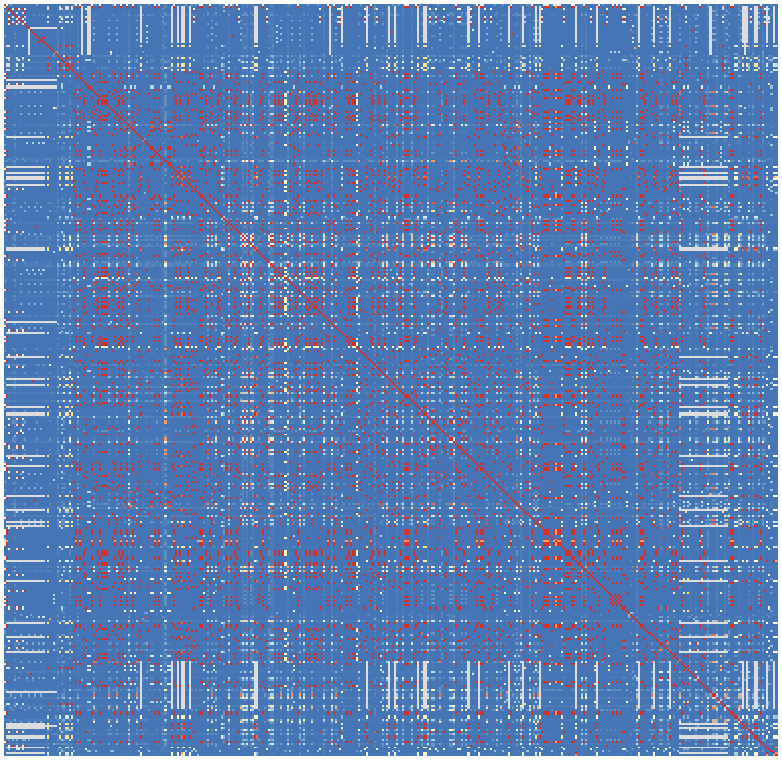

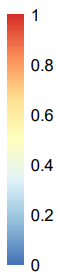


A.

B.

r^2^ value

**Figure S4.** Examining the effect of introgression blocks on linkage disequilibrium. Both A and B show a heatmap of r^2^ between marker pairs along chromosome 02 at a spacing of approximately 250 kb. The individuals with introgression blocks in the WGS population have been masked in part B while no masking was done for part A.


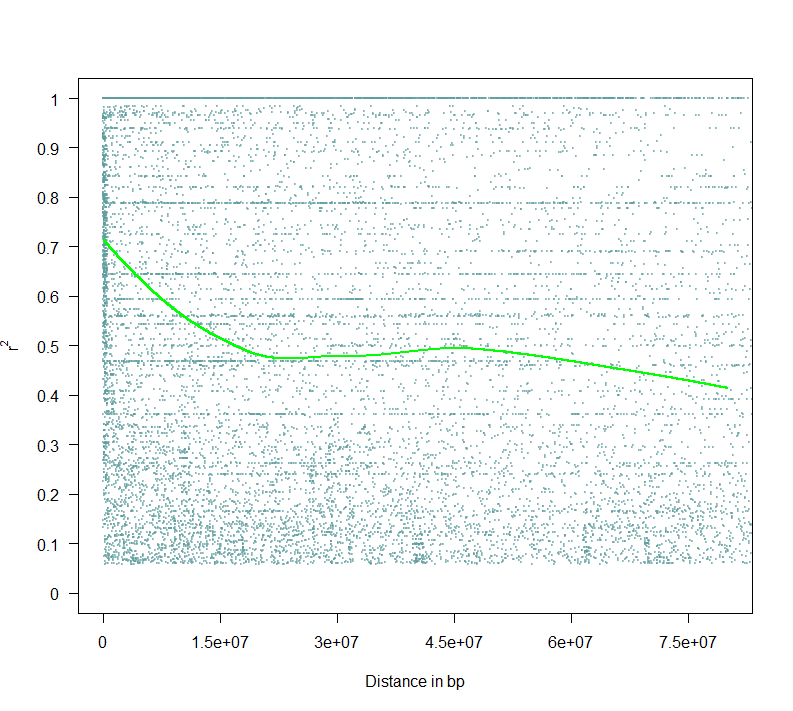


**Figure S5**. Linkage Disequilibrium decay chart across the whole genome. Visualizing the decline of r^2^ (y-axis) over physical genomic distance (x-axis). The loess curve is shown in bright green.

**Supplementary Files 1-5.** VCF files 1-4 included here contain the positions of the markers in Sets 1-4 respectively. Supplementary file 5 contains the positions of all Indels filtered out in the formation of Marker Set 1. All coordinates are based on the Bailey II genome.

# **Supplementary Text**

**Note S1.** Marker Discovery Pipeline Variation on Bailey II Unplaced Scaffolds

Variation detected through the marker discovery pipeline was described in the main manuscript without unplaced scaffolds. Here, variation across the unplaced scaffolds is made available. From the raw output of the GATK joint calling process (Poplin et al., 2018), 30,884 variants were present on the 406 unplaced scaffolds of Bailey II.

**Note S2.** Delimit and Design markers for *A. cardenasii* Introgressions Materials

Lines GP-NC WS 5, GP-NC WS 7, GP-NC WS 8, GP-NC WS 9, GP-NC WS 10, GP-NC WS 11, GP-NC WS 12, GP-NC WS 14 and GP-NC WS 15 were added for the sole purpose of capturing *A. cardenasii* introgression blocks from germplasm released from the NCSU hexaploid route. These lines are not central to the NCSU Virginia-Type peanut breeding program and therefore were not included in the WGS panel. All cultivation, library preparation, sequencing, and downstream bioinformatics of these nine GP-NC WS lines were conducted in the same way as described for the WGS panel. WGS panel genotype data in conjunction with the nine GP-NC WS genotype data were used to identify *A. cardenasii* introgressions and inform PACE marker development. When considering the presence/absence of blocks, individuals which were heterozygous for a block were counted towards presence.

**Note S3.** High-Quality Marker Set for Future GS Genotyping

Markers were selected from Set 1 and Set 3, to develop marker Set 4 for future use in a mid-density genotyping approach. Command line utilities were used to create a bed file of regions 25-145 base pairs on either side of the markers- as these are the regions where probes may be designed (internal communication). These flanking regions were intersected with repetitive regions of the Bailey II genome (as annotated previously by RepeatMasker v. 4.1.2 (Smit et al., 2013)) with BEDtools (Quinlan & Hall, 2010). If the regions flanking the markers had an overlap of more than 20 bp, that region was marked as failed. If a marker had a fail on both the right hand and left hand sides it was removed from the pool of candidate markers. Next markers were selected for inclusion in Set 4 based on their membership in a category. The categories were A) previous research, B) PACE assay, C) haplotype block,  D) positional, E) manual duplication, F) exonic, and G) multiple. Category A pertains to informative markers from the preliminary work done with the Axiom Arachis2 array (Hancock, 2018). Category B pertains to the *A. cardenasii* introgression PACE markers developed in this study. Category C were markers within and between haplotype blocks. Five markers were included from each side of the haplotype blocks along with three markers from the center of the haplotype blocks. A maximum of four markers were included from the areas between neighboring haplotype blocks. Haplotype blocks were computed by plink v. 1.9 (Chang et al., 2015) with a window of 500 kb using the candidate markers described above after manually masking introgressions. Category D were markers placed approximately every 1 Mb across the Bailey II genome. Category E represents a random selection of markers from marker Set 3. Category F were markers within annotated exons. Some markers qualified for inclusion in two or more categories (Category G).
